# NCX2 Targeting as a Neuroprotective Strategy Against Oxaliplatin-Induced Peripheral Neurotoxicity

**DOI:** 10.3390/cancers18183024

**Published:** 2026-09-17

**Authors:** Sara Di Girolamo, Chiara Invernizzi, Margherita Francesca Kraus, Elisa Ballarini, Alessio Malacrida, Mario Mauri, Virginia Rodriguez-Menendez, Stephen N. Housley, Paola Alberti

**Affiliations:** 1 Experimental Neurology Unit, School of Medicine and Surgery, 20900 Monza, Italy; sara.digirolamo@unimib.it (S.D.G.); c.invernizzi14@campus.unimib.it (C.I.); elisa.ballarini@unimib.it (E.B.); alessio.malacrida@unimib.it (A.M.); mario.mauri@unimib.it (M.M.); virginia.rodriguez1@unimib.it (V.R.-M.); 2 PhD Program in Neuroscience, University of Milano-Bicocca, 20900 Monza, Italy; 3 S.C. Neuroscienze, Istituto Zooprofilattico Sperimentale del Piemonte Liguria e Valle D’Aosta, 10154 Torino, Italy; margheritafrancesca.kraus@izsplv.it; 4 School of Biological Sciences, Georgia Institute of Technology, Atlanta, GA 30332, USA; nickhousley@gatech.edu

**Keywords:** oxaliplatin-induced peripheral neuropathy, oxaliplatin-induced peripheral neurotoxicity, NCX, NCX2, axonal damage, neuropathy, axonal hyperexcitability

## Abstract

**Background/Objectives**: Oxaliplatin (OHP) is a key component of colorectal cancer chemotherapy, but OHP-induced peripheral neurotoxicity (OIPN) is a major dose-limiting adverse effect. OHP-related sodium-channel dysfunction could increase intracellular Na^+^ and sustain depolarization, conditions that may theoretically favor reverse-mode NCX activity, in which the exchanger promotes Ca^2+^ entry rather than Ca^2+^ extrusion. This study evaluated whether NCX2 targeting could reduce OHP-induced sensory neuron injury. **Methods**: Primary mouse dorsal root ganglion neurons were exposed to OHP, with or without SEA0400 (NCX modulator) pretreatment or siRNA-mediated NCX2 knockdown. Neuronal viability, neurite elongation, live-cell morphology, NCX2 fluorescence, and lipid droplet accumulation were quantified. **Results**: OHP reduced neuronal survival and neurite elongation and induced stress-related morphological changes and lipid droplet accumulation. NCX2-associated fluorescence varied by neuronal subtype, dose, and time. SEA0400 and partial NCX2 knockdown attenuated selected injury endpoints, mainly under low-dose conditions. **Conclusions**: These findings suggest that NCX2 may be involved in selected OHP-induced sensory neuron injury endpoints and support further investigation of NCX2 as a potential preventive target. However, reverse-mode NCX activity, Na^+^/Ca^2+^ fluxes, NCX2 directionality, and translational relevance require direct validation.

## Simple Summary

Oxaliplatin is an important chemotherapy drug, but it can damage sensory nerves and cause lasting numbness, neuropathic pain, and manual dexterity/gait instability. This study explored whether blocking a nerve-cell transporter called NCX, especially its NCX2 form, could help protect nerve cells from oxaliplatin-related injury. Using in vitro models, the researchers showed that oxaliplatin reduced nerve-cell survival, shortened nerve fibers, and triggered signs of cellular stress. A drug that inhibits NCX activity, SEA0400, and targeted reduction of NCX2 both lessened these harmful effects, particularly under lower oxaliplatin exposure. These findings suggest that NCX2 targeting may help reduce oxaliplatin-induced injury in this experimental model and warrants further study.

## 1. Introduction

Oxaliplatin (OHP), a third-generation platinum-based chemotherapeutic agent, is a cornerstone of current adjuvant and metastatic colorectal cancer regimens [1,2]. Despite its robust antitumor efficacy, the clinical utility of OHP is limited by oxaliplatin-induced peripheral neurotoxicity (OIPN), a frequent and potentially disabling adverse effect that may require dose reduction, treatment delay, or premature discontinuation, thereby compromising therapeutic outcomes [3,4]. OIPN presents with both acute and chronic manifestations. Acute OIPN typically develops during or within hours after infusion and is characterized by cold-aggravated paresthesia, dysesthesia in the distal extremities and perioral region, jaw stiffness, and painful muscle cramps [5]. These symptoms are generally transient but clinically relevant because they reflect early functional alterations in sensory axons.

Chronic OIPN, in contrast, is a cumulative and dose-dependent sensory polyneuropathy that emerges after repeated treatment cycles. It manifests as symmetrical stocking-and-glove sensory loss, neuropathic pain, and sensory ataxia due to proprioceptive impairment [6,7,8]. Symptoms may persist long after chemotherapy completion and can even worsen for several months after treatment withdrawal, a phenomenon known as coasting [9]. Importantly, clinical and preclinical evidence indicates that acute OIPN may correlate with subsequent chronic sensory axonopathy, suggesting a possible association between early functional hyperexcitability and later structural axonal degeneration [10,11,12,13,14].

A key requirement for normal sensory neuron function is the tight regulation of intracellular sodium and calcium concentrations. Sodium gradients sustain action potential generation and propagation, whereas calcium acts as a central signaling ion controlling excitability, mitochondrial activity, cytoskeletal dynamics, and cell survival [15,16]. Because excessive intracellular calcium can activate degenerative pathways, neurons rely on several buffering and extrusion systems to maintain calcium homeostasis. Among these, the sodium–calcium exchanger (NCX) is a major plasma membrane transporter that couples sodium and calcium movement across the membrane [17,18,19]. Under physiological conditions, NCX generally operates in forward mode, extruding calcium in exchange for sodium entry. However, when intracellular sodium rises and the membrane remains depolarized, NCX can operate in reverse mode, extruding sodium while promoting calcium influx [17,20,21,22].

This bidirectional behavior may provide a possible framework for hypothesizing a relationship between OHP-induced acute hyperexcitability and chronic axonal damage (AxD). Acute OIPN is mainly attributed to alterations in voltage-gated sodium channels (NaV), particularly the tetrodotoxin-sensitive NaV1.6 and tetrodotoxin-resistant NaV1.8 isoforms expressed in dorsal root ganglion (DRG) sensory neurons [23,24]. OHP delays NaV inactivation and markedly enhances both resurgent and persistent sodium currents, leading to intracellular sodium accumulation and sustained membrane depolarization: under these conditions, the electrochemical gradient may favor reverse-mode NCX activity, potentially resulting in pathological calcium entry into axons and neuronal soma [17,20,21,25].

Calcium overload is recognized as a central driver of axonal degeneration. Excess calcium can activate deleterious downstream cascades, including calpain activation, mitochondrial permeability transition, autophagic stress, and broader cell-death-associated signaling that may include necroptosis-related pathways, ultimately promoting neurite fragmentation and neuronal death [26,27]. In this framework, OHP-induced sodium channel dysfunction could initiate axonal hyperexcitability, whereas reverse-mode NCX activity may amplify injury by converting sodium overload into calcium-dependent structural damage.

If NCX-mediated calcium entry contributes to OHP-induced neuronal injury, then limiting NCX activity could provide a rationale for testing NCX inhibition as a potential preventive neuroprotective strategy. In previous work, we showed that topiramate, by modulating neuronal excitability, prevents oxaliplatin-related axonal hyperexcitability and attenuates OHP-induced peripheral neurotoxicity in a well-characterized rat model [14]. We also provided preliminary evidence that pharmacological NCX inhibition with SEA0400 is associated with neuroprotective effects and supports a potential role for sodium–calcium exchanger isoform 2 (NCX2) in OHP-induced axonal damage [25]. Nevertheless, the contribution of individual NCX isoforms, and particularly NCX2 (SLC8A2), which is highly expressed in the mammalian peripheral nervous system [23], remains incompletely defined.

The present study was therefore designed to evaluate whether NCX inhibition, and more specifically NCX2 targeting, is associated with reduced OHP-induced injury in sensory neurons. To address this aim, we used primary mouse DRG sensory neuron cultures combined with 3D label-free holotomographic live-cell imaging to longitudinally monitor OHP-induced cellular injury. Specifically, we assessed the dose- and time-dependent effects of OHP on neuronal survival and neurite elongation; characterized real-time morphological features compatible with cellular stress through refractive index signatures, including vacuolar structures compatible with autophagic vacuolization, morphology-inferred necroptosis-like degeneration, and lipid droplet accumulation; and evaluated the potential preventive neuroprotective effects of NCX inhibition using the pharmacological blocker SEA0400 [28,29,30,31,32] and genetic knockdown of NCX2 by small interfering RNA.

## 2. Materials and Methods

### 2.1. Animals

C57BL/6J male mice aged 7–8 weeks were purchased from Envigo Laboratory (Udine, Italy). Animals were housed in temperature- and humidity-controlled rooms maintained at 22 ± 2 °C and 55 ± 10% relative humidity under a 12 h light/dark cycle, with ad libitum access to food and water. Clinical conditions were monitored daily. All experimental procedures were performed in accordance with institutional guidelines and complied with national legislation (D. L.vo 26/2014, Gazzetta Ufficiale della Repubblica Italiana, n. 61, 14 March 2014) and international laws and policies, including European Union Directive 2010/63/EU and the Guide for the Care and Use of Laboratory Animals, U.S. National Research Council, 1996. Experiments were approved by the Italian Ministry through project notification NOT FB7CC.N.2JB and NOT FB7CC.N.IF6 and by the University of Milano-Bicocca under institutional permission number 11/2024 and 07/2026.

### 2.2. Isolation, Culture, and Treatment of Mouse DRG Neurons

C57BL/6J mice were euthanized by CO_2_ overdose inhalation. Dorsal root ganglia (DRG) were aseptically removed and cultured. Briefly, DRG from cervical to sacral levels, up to S2, were bilaterally excised, collected, and carefully desheathed in a dish containing cold Ham’s F-12 nutrient mixture medium (EuroClone S.p.A., Milan, Italy). After incubation for 1.25 h at 37 °C with collagenase from Clostridium histolyticum (Merck Life Science S.r.l., Milan, Italy) and DNase I (Invitrogen, Thermo Fisher Scientific, Waltham, MA, USA), DRG were dissociated. Cells were plated on laminin-coated 35 mm dishes (Merck Life Science S.r.l., Milan, Italy) and maintained in a humidified 5% CO_2_ incubator at 37 °C in Ham’s F-12 and low-glucose DMEM-based medium (EuroClone S.p.A., Milan, Italy). The media were enriched with L-glutamine (1X, EuroClone S.p.A., Milan, Italy), Penicillin-Streptomycin (EuroClone S.p.A., Milan, Italy), B-27^®^ Supplement Minus AO (50X, Gibco, Thermo Fisher Scientific, Waltham, MA, USA) and Neurobasal Medium (1X, Gibco, Thermo Fisher Scientific, Waltham, MA, USA).

### 2.3. Cellular Treatments and Neurotoxicity Assessment

**Figure A1:**
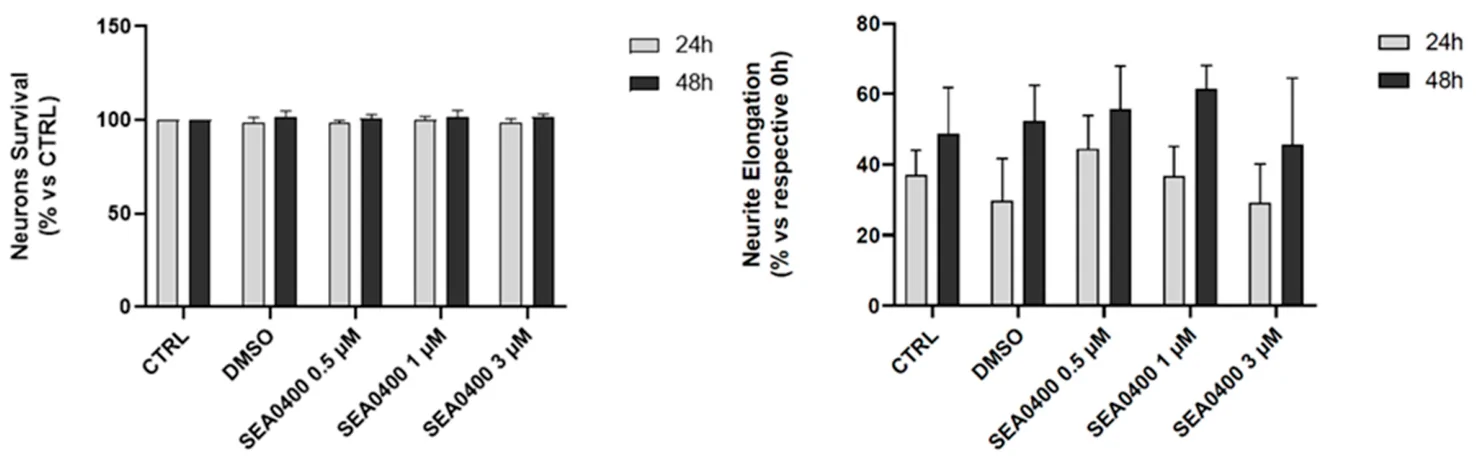
Effects of SEA0400 exposure on DRG neuron viability and neurite elongation. Histograms show neuronal survival and neurite length in CTRL cultures, DMSO-treated cultures, and cultures exposed to SEA0400 at 0.5, 1, or 3 µM for 24 or 48 h. SEA0400 did not significantly affect neuronal viability or neurite elongation compared with CTRL. Data are presented as mean ± SEM. Statistical analysis was performed using one-way ANOVA followed by Tukey’s post hoc test vs. CTRL.

**Figure A2:**
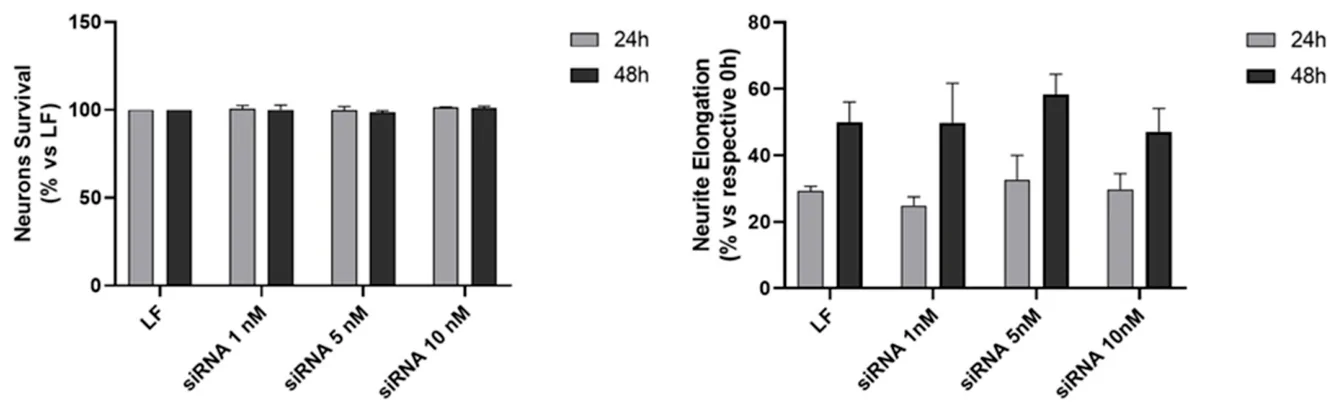
Effects of siRNA transfection on DRG neuron viability and neurite elongation. Histograms show neuronal survival and neurite length in vehicle-treated cultures and in cultures transfected with NCX2-targeting siRNA at 1, 5, or 10 pmol. siRNA transfection did not significantly affect neuronal viability or neurite elongation at 24 or 48 h compared with vehicle-treated cultures. Data are presented as mean ± SEM. Statistical analysis was performed using one-way ANOVA followed by Tukey’s post hoc test vs. vehicle control.

To assess the neurotoxic effects of oxaliplatin (OHP; Selleckchem Chemicals, GmbH, Cologne, Germany), DRG neurons were exposed to different drug concentrations (7.5, 15, 25, and 50 µM) for 48 h. This treatment was started 48 h after DRG isolation. OHP was dissolved in sterile water to obtain a stock solution and freshly diluted in culture medium to the required experimental concentrations. To investigate the neuroprotective effects of SEA0400 (SML2054, Merck Life Science S.r.l., Milan, Italy), a potent NCX inhibitor, DRG neurons were pretreated with SEA0400 (1 µM, 3 h) before exposure to OHP (7.5 or 25 µM). SEA0400 was dissolved in DMSO to obtain a stock solution and freshly diluted in culture medium. NCX2 expression was silenced using Ambion Silencer Select predesigned siRNAs (4390816, ID: s99928; Ambion by Life Technologies, Monza, Italy); the 5 pmol siRNA dose used for knockdown experiments was selected according to the manufacturer’s datasheet and confirmed by preliminary dose-finding experiments. Lipofectamine 2000 (11668030, Invitrogen, Thermo Fisher Scientific, Waltham, MA, USA) was used as the transfection reagent, and DRG neurons were incubated with the siRNA–lipid complex for 24 h before 48 h OHP exposure. Preliminary dose-finding experiments confirmed that SEA0400 exposure and siRNA transfection did not intrinsically affect DRG neuron viability or neurite elongation, as reported in Appendix A (Figure A1 and Figure A2). The peripheral neurotoxic effects of OHP were investigated in vitro using an established DRG explant model from C57BL/6J male mice, with neurite elongation and neuronal viability as primary readouts of neurotoxicity [33]. Neurotoxicity was evaluated by counting living neurons and measuring DRG neurite length at 0, 24, and 48 h after treatment using ImageJ software, version 1.54p (NIH, Bethesda, MD, USA); for neurotoxicity assessment, “0 h” was defined as the time at which OHP was added to the medium, corresponding to 48 h after plating. Changes in neurite length at 24 and 48 h after OHP administration were expressed as percentage relative to 0 h in the same experiment.

### 2.4. 3D Holotomographic Live Cell Imaging

This live imaging technique relies on the detection of the refractive index (RI). The RI is a physical quantity that describes the extent to which light slows down when passing through a material compared with its speed in a vacuum. In biological cells, different cellular structures have distinct compositions and densities and therefore exhibit different RIs. Imaging techniques that measure the RI can consequently provide information on cellular structure, composition, and mass distribution without necessarily requiring dyes or fluorescent labels. The RI-based 3D holotomographic images were acquired using a 3D Cell Explorer 96focus microscope (Nanolive SA, Tolochenaz, Switzerland) equipped with a 520 nm laser for tomographic phase microscopy. Laser irradiance was set to 0.2 nW/µm^2^, acquisition time was 45 ms per frame, and field depth was 30 µm. The system included a Basler ace acA1920-155um camera and an air objective lens (NA = 0.8, 60× magnification). Holographic acquisition was enhanced by rotational scanning using a rotating mirror, which reduces coherent diffraction noise. In combination with an automated stage and laser-based autofocus, the microscope enabled stable long-term acquisitions. The system was equipped with an Okolab incubation chamber controlling temperature (37 °C), humidity (90%), and gas composition (5% CO_2_). DRG neurons were seeded in 35 mm imaging dishes (IBIDI, Martinsried, Germany) on laminin-coated glass in Ham’s F-12 and low-glucose DMEM-based medium and placed inside the top-stage incubator. Imaging acquisition was initiated 24 h after OHP treatment and continued for up to 52 h after treatment initiation. All time points from time-lapse experiments were reconstructed and stored as 3D volumes using Eve software (version 2.2.1.2207), which controls the microscope and manages initial data processing.

Lipid droplet analysis—Nanolive Eve Analytics software (version 2.2.1.2207): Lipid droplets are specialized cellular organelles involved in the storage and metabolism of lipids and exhibit refractive index (RI) features that can support their label-free identification when combined with morphology-based criteria. Therefore, we performed lipid droplet analysis on reconstructed RI tomograms acquired with the 3D Cell Explorer 96focus system. All time-lapse datasets were reconstructed using Nanolive Eve software and analyzed using the Smart Lipid Droplet Assay of Eve Analytics software. Lipid droplets were identified in live DRG cells as discrete, round intracellular structures displaying higher RI values than the surrounding cytoplasm, consistent with the optical properties of lipid-rich organelles in label-free holotomographic imaging. For each experimental condition, cell bodies and neurites were selected as regions of interest. Lipid droplets within each selected cell were segmented from RI volumes using RI-based contrast and object morphology criteria, following label-free lipid droplet visualization workflows previously described for 3D holotomographic microscopy with Nanolive [34]. Quantification was performed at the single-cell level and expressed as lipid droplet count per cell. The same segmentation parameters and quality-control criteria were applied across all treatment groups within each experiment. For time-lapse acquisitions, lipid droplet counts were extracted at the indicated time points after treatment, with the 52 h time point used for the comparative analyses shown in the Results. Data from independent fields and cultures were pooled by condition and used for statistical analysis as described below.

### 2.5. Immunofluorescence

DRG neurons were fixed in 10% formalin at 24 and 48 h after OHP treatment. After washing in PBS, neurons were incubated with 0.3 M glycine for 10 min and then, without washing, blocked with 3% bovine serum albumin (BSA) and 3% normal donkey serum (NDS) in PBS for 1 h 30 min at room temperature. Primary antibodies against NCX2 (ANX-012, Alomone Labs, Jerusalem, Israel, 1:200) and NF200 (N2912, Merck Life Science S.r.l., Milan, Italy, 1:500) were diluted in 1% BSA and 1% NDS in PBS and incubated overnight at 4 °C. Negative-control samples were processed in parallel by omitting the primary antibodies and incubating the cultures with secondary antibodies only. These controls were used to assess background staining, verify immunofluorescence signal specificity, and exclude relevant non-specific secondary-antibody staining. The following day, a 1% BSA and 1% NDS solution was used for washing before incubation with secondary antibodies (Donkey anti-rabbit 555, Invitrogen Thermo Fisher Scientific, Waltham, MA, USA, 1:200; Goat anti-mouse 647, Cell Signaling Technology, Danvers, MA, USA, 1:200) diluted in PBS for 1 h at room temperature. Isolectin GS-IB4 (I21411, Invitrogen, Thermo Fisher Scientific, Waltham, MA, USA, 1:200) was added together with the secondary antibodies. Subsequent washes were performed with PBS. Slides were mounted with Fluoroshield (F6057, Merck Life Science S.r.l., Milan, Italy), which contains DAPI for nuclear staining. Images were acquired using a widefield microscope (Zeiss Axio Observer, ZEISS Microscopy GmbH, Jena, Germany) with a 20× objective, and background noise was removed using the negative-control samples.

Fluorescence images were analyzed using ZEISS Arivis Cloud and ZEISS Arivis Pro software (ZEISS Microscopy GmbH, Jena, Germany). Neuronal cell bodies were identified and segmented using the AI-based segmentation module available in ZEISS Arivis Cloud. Neurons were then classified according to their molecular phenotype as IB4-positive or NF200-positive. Neuronal classification was performed using a custom analysis pipeline in ZEISS Arivis Pro (ZEISS Microscopy GmbH, Jena, Germany). For each segmented neuronal cell body, the fluorescence intensity corresponding to the IB4 or NF200 signal was measured. Positivity was assigned when the fluorescence signal exceeded a predefined intensity threshold established to distinguish specific staining from background fluorescence.

Following classification, mean NCX2 fluorescence intensity was quantified for each segmented neuron. To account for differences in fluorescence intensity between experimental acquisitions, NCX2 values were normalized to the corresponding control group.

NCX2 expression was first compared between control and OHP-treated groups separately within the IB4-positive and NF200-positive neuronal populations. Subsequently, NCX2 fluorescence intensity was compared between IB4-positive and NF200-positive neurons within each treatment group, allowing the assessment of potential differences in NCX2 expression between the two neuronal subpopulations following OHP exposure.

### 2.6. NCX2 Quantitative PCR Validation

Quantitative real-time PCR (qPCR) was performed to validate NCX2 silencing after siRNA transfection. Total RNA was extracted from DRG primary neuronal cultures using TRIzol^®^ Reagent (Ambion, Life Technologies, Monza, Italy). RNA concentration and purity were evaluated spectrophotometrically using a NanoDrop™ 2000 spectrophotometer (Thermo Fisher Scientific, Wilmington, DE, USA). Samples with an A260/A280 ratio between 1.5 and 2.0 were used for subsequent analyses. Reverse transcription was performed using LunaScript^®^ RT SuperMix 5× (New England Biolabs, Ipswich, MA, USA) and a Mastercycler^®^ nexus X2 PCR Thermal Cycler (Eppendorf, Hamburg, Germany), starting from 100 ng of RNA for each sample. qPCR was performed using Luna^®^ Universal qPCR Master Mix 2× (New England Biolabs, Ipswich, MA, USA) on a QuantStudio 7 Real-Time PCR System (Applied Biosystems, Thermo Fisher Scientific, Waltham, MA, USA). Each reaction was carried out in a total volume of 10 µL containing 5 µL of Master Mix, 0.5 µM of each primer, 2 µL of diluted cDNA, and nuclease-free water. The target gene was assessed using TaqMan^®^ Gene Expression Assay Mm0455836_m1 (Applied Biosystems, Thermo Fisher Scientific, Waltham, MA, USA) for Slc8a2, while the housekeeping gene was assessed using TaqMan^®^ Gene Expression Assay Mm99999915_g1 (Applied Biosystems, Thermo Fisher Scientific, Waltham, MA, USA) for Gapdh. Thermal cycling conditions were as follows: initial denaturation at 50 °C for 2 min and 95 °C for 10 min, followed by 40 cycles of denaturation at 95 °C for 15 s and annealing/extension at 60 °C for 1 min. A melt curve analysis was performed at the end of each run to verify amplification specificity. Relative gene expression levels were normalized to GAPDH expression and calculated using the 2^−ΔΔCt^ method (Livak and Schmittgen 2001, PMID: 11846609) [35].

### 2.7. Statistical Analyses

All experiments were performed using three independent biological replicates (*n* = 3). Each biological replicate consisted of a primary DRG neuronal culture prepared from DRG isolated from one adult C57BL/6J mouse; therefore, the independent DRG preparation obtained from an individual animal was considered the biological replicate and experimental unit. Dishes, imaging fields, individual neurons, neurites, and cells analyzed within the same culture were considered technical observations nested within the corresponding biological replicate and were not interpreted as independent biological replicates. Where possible, individual data points were plotted together with summary statistics. Statistical analyses were performed using GraphPad Prism (version 8.0.2; GraphPad Software, San Diego, CA, USA) and R (version 4.6.1). Neurite elongation and neuronal survival data were normally distributed; therefore, comparisons for these endpoints were performed using one-way ANOVA followed by Tukey’s post hoc test. This approach was retained to ensure consistency with the established DRG neurotoxicity methodology routinely used and previously validated in our laboratory. Nanolive lipid droplet count data were analyzed using two-way ANOVA to account for treatment condition, time, and their interaction during longitudinal imaging experiments. To quantify changes in mean fluorescence intensity (MFI), generalized linear mixed-effects modeling was performed in R using the glmer function from the lme4 package. MFI values were modeled as the dependent variable, with cell type, OHP dose level, and time included as fixed factors together with all two-way and three-way interaction terms (cell type × OHP dose level × time). To account for biological and technical variability between independent assays, experimental run was included as a random intercept (1 | experiment). Continuous MFI data were specified using a Gamma distribution with a log-link function, or log-transformed before modeling when required, to improve residual normality and homoscedasticity. Statistical significance of main effects and interaction terms was evaluated using Type III Analysis of Variance Wald chi-square tests with the car package. Post hoc pairwise comparisons across cell types, doses, and time points were calculated using estimated marginal means with the emmeans package and Tukey’s honest significant difference adjustment for multiple testing. Candidate models of varying complexity, ranging from main-effects-only to full higher-order interaction structures, were evaluated using Akaike Information Criterion (AIC) and Bayesian Information Criterion (BIC). For fixed-effect model comparisons, candidate structures were fitted using Maximum Likelihood estimation. Lower AIC and BIC values were considered to indicate a better balance between model fit and parsimony, and differences greater than 2 relative to the lowest-value model were considered meaningful. When AIC and BIC favored different structures, the more parsimonious model supported by BIC was prioritized to reduce overfitting. Once the final model structure was established, linear models were refitted using Restricted Maximum Likelihood for final parameter estimation and hypothesis testing. All tests were two-tailed, and statistical significance was set at α = 0.05.

## 3. Results

### 3.1. Chronic OHP Exposure Alters DRG Neuron Viability and Neurite Elongation in a Dose- and Time-Dependent Manner

**Figure 1:**
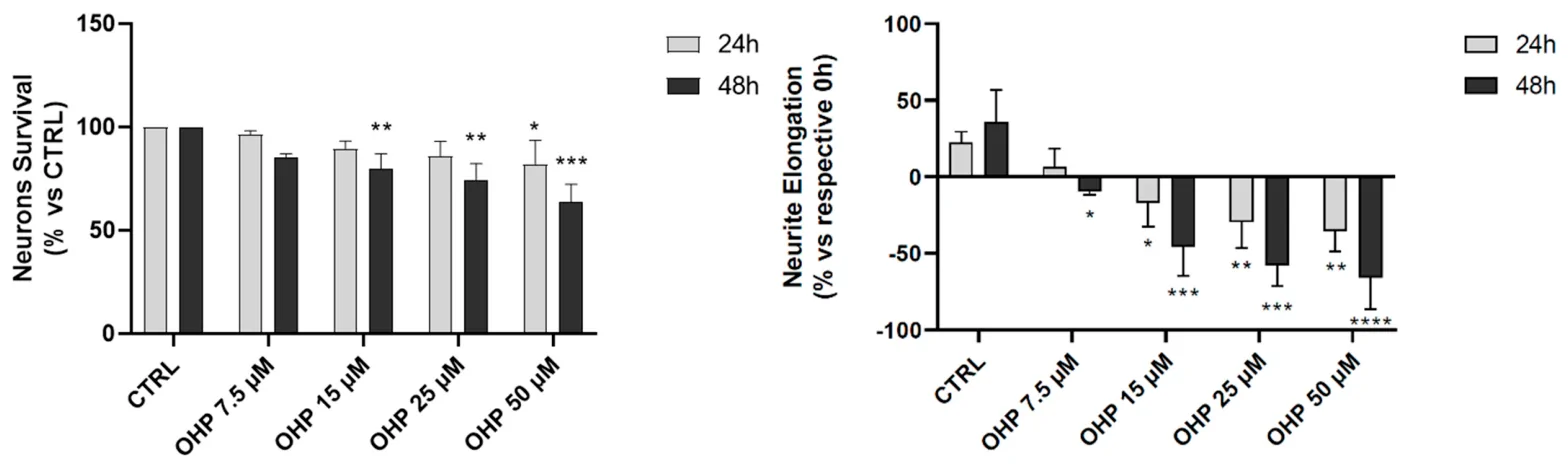
Effects of chronic OHP exposure on DRG neuron viability and neurite elongation. Histograms show neuronal survival and neurite length in control cultures and in cultures exposed to OHP at 7.5, 15, 25, or 50 µM for 24 or 48 h after treatment. OHP was associated with dose- and time-dependent reductions in neurite elongation and neuronal viability. Data are presented as mean ± SEM. Statistical analysis was performed using one-way ANOVA followed by Tukey’s post hoc test; significance values indicate comparisons with untreated control cultures, not comparisons between 24 h and 48 h: * *p* < 0.05, ** *p* < 0.01, *** *p* < 0.001, and **** *p* < 0.0001 vs. CTRL.

As shown in Figure 1, control DRG neurons maintained in culture medium extended long neurites, whereas exposure to OHP for 24 and 48 h induced progressive neurite shortening. Both neurite impairment and loss of viability followed a dose- and time-dependent pattern. Exposure to 7.5 µM OHP did not significantly affect neurite length at 24 h, whereas a 10% reduction was observed at 48 h (*p* < 0.05 vs. CTRL), without significant changes in viability. At 15 µM, OHP reduced neurite length by 15% at 24 h (*p* < 0.05 vs. CTRL) and by 45% at 48 h (*p* < 0.001 vs. CTRL), while viability was significantly reduced only at 48 h (20% decrease; *p* < 0.01 vs. CTRL). Exposure to 25 µM OHP induced 30% and 60% neurite shortening at 24 and 48 h, respectively (*p* < 0.01 and *p* < 0.001 vs. CTRL), with a 25% reduction in viability at 48 h (*p* < 0.01 vs. CTRL). The highest concentration, 50 µM, produced the most pronounced effects, with 35% neurite shortening at 24 h (*p* < 0.01 vs. CTRL), 65% shortening at 48 h (*p* < 0.0001 vs. CTRL), and viability reductions of 20% and 40% at 24 and 48 h, respectively (*p* < 0.05 and *p* < 0.001 vs. CTRL).

### 3.2. 3D Holotomographic Live-Cell Imaging Reveals OHP-Induced DRG Morphological Injury Through Refractive Index Signatures

**Figure 2:**
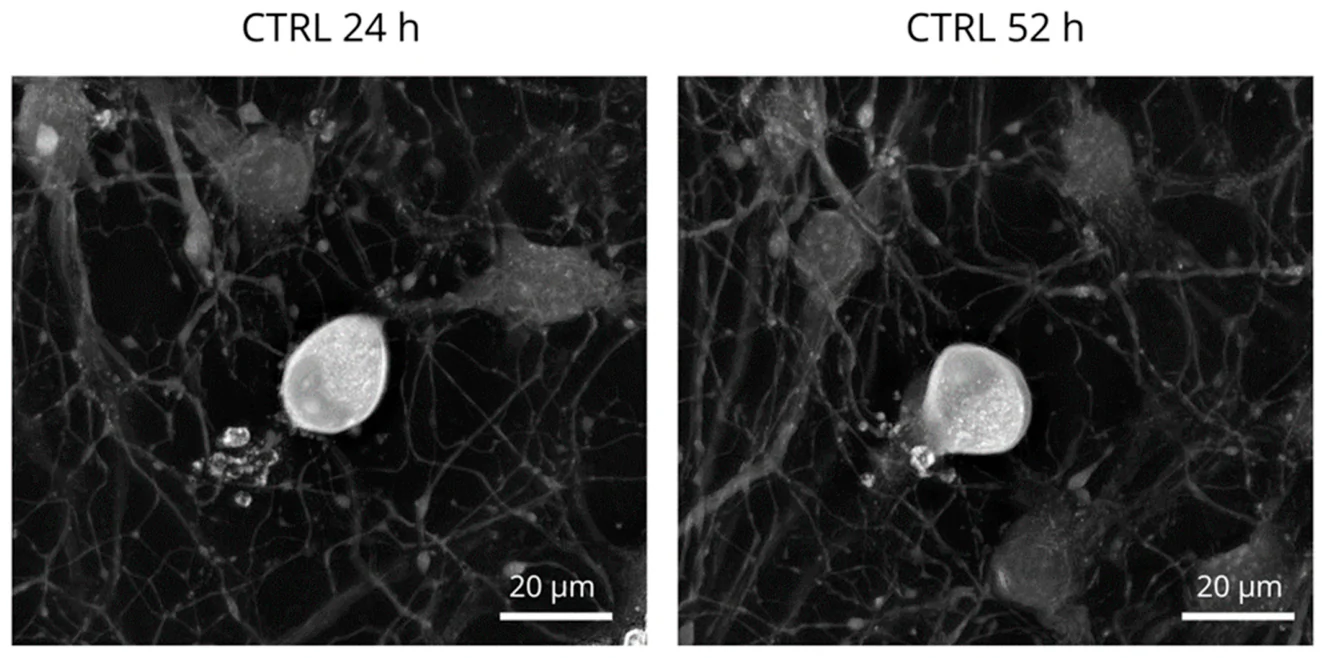
Live-cell holotomographic imaging of DRG neurons in CTRL condition. Representative images acquired with the Nanolive 3D Cell Explorer 96focus microscope show primary adult mouse DRG neuronal cultures under untreated control conditions. Refractive index signatures show a representative viable neuron with preserved cell body and neurite morphology, along with an increase in the number of surrounding support cells. Images were acquired using a 5 × 5 grid scan, with each grid measuring 90 × 90 µm, every 15 min for 28 h at 7 fps, from 24 to 52 h after treatment initiation in the corresponding experimental timeline.

**Figure 3:**
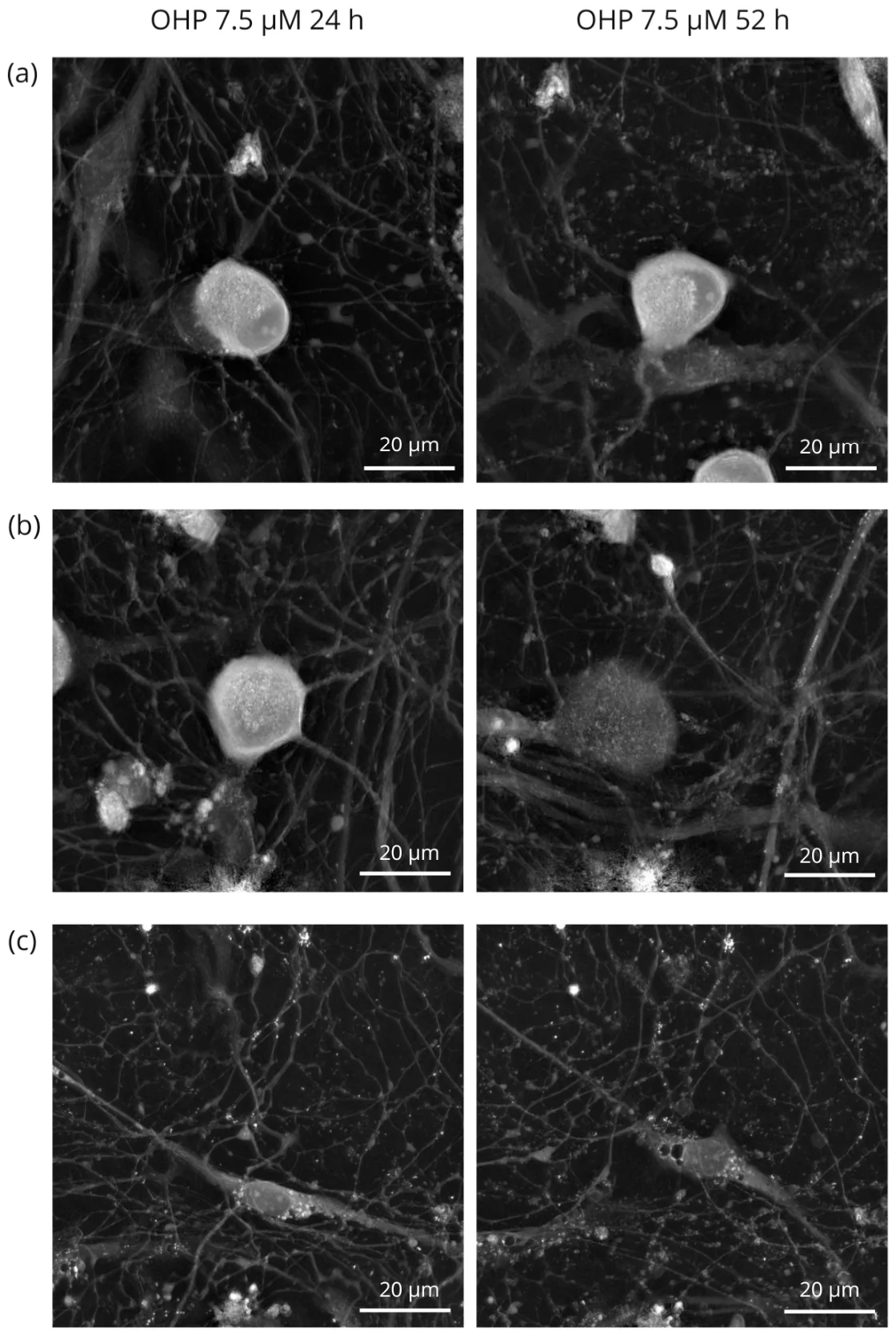
Live-cell holotomographic imaging of DRG neurons exposed to low-dose OHP. Representative images acquired with the Nanolive 3D Cell Explorer 96focus microscope show primary adult mouse DRG neuronal cultures after exposure to 7.5 µM OHP. Refractive index signatures revealed neurite fragmentation and degeneration (**a**), morphology-inferred necroptosis-like degeneration (**b**), and vacuolar structures compatible with autophagic vacuolization (**c**). Images were acquired using a 5 × 5 grid scan, with each grid measuring 90 × 90 µm, every 15 min for 28 h at 7 fps, from 24 to 52 h after treatment initiation.

**Figure 4:**
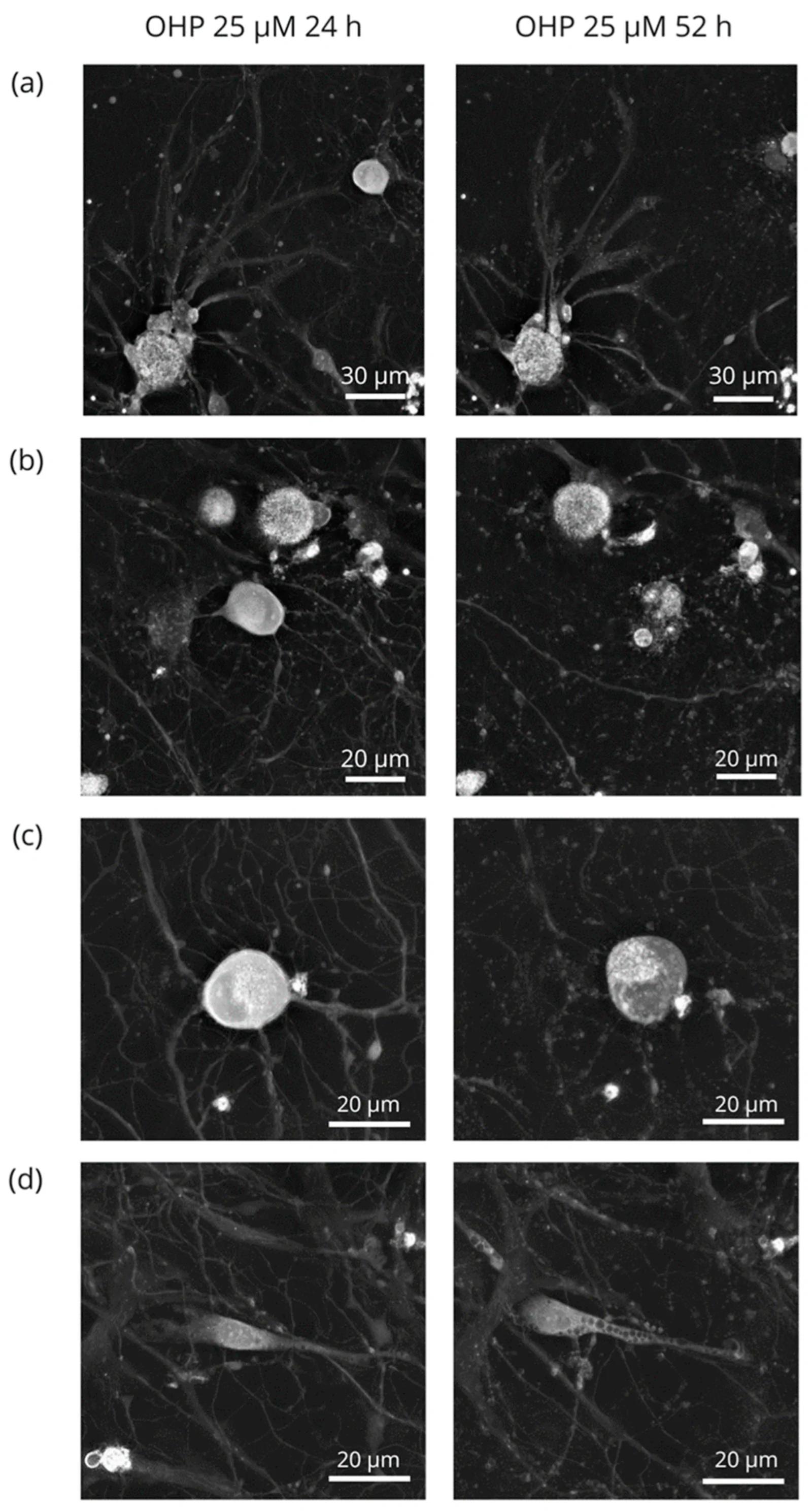
Live-cell holotomographic imaging of DRG neurons exposed to high-dose OHP. Representative images acquired with the Nanolive 3D Cell Explorer 96focus microscope show primary adult mouse DRG neuronal cultures after exposure to 25 µM OHP. Refractive index signatures revealed neurite fragmentation and degeneration (**a**,**b**), morphology-inferred necroptosis-like degeneration (**c**), and vacuolar structures compatible with autophagic vacuolization (**d**). Images were acquired using a 5 × 5 grid scan, with each grid measuring 90 × 90 µm, every 15 min for 28 h at 7 fps, from 24 to 52 h after treatment initiation.

To investigate OHP-induced morphological alterations by live-cell imaging, DRG neurons from C57BL/6J male mice were analyzed using the Nanolive 3D holotomographic microscope and compared under untreated control and OHP-exposed conditions. Untreated control cultures were first monitored over time to provide a reference for morphology during image acquisition (Figure 2). Low-dose OHP exposure (7.5 µM; Figure 3) and high-dose OHP exposure (25 µM; Figure 4) were then monitored for up to 52 h. This platform provides high-content refractive index (RI) images capable of capturing fine cellular structures and dynamic morphological changes at subcellular resolution (<200 nm). Compared with untreated control cultures, OHP-exposed cells displayed widespread neurite fragmentation and degeneration after both 7.5 µM and 25 µM exposure (Figure 3a and Figure 4a,b), together with morphology-inferred necroptosis-like degeneration (Figure 3b and Figure 4c) and vacuolar structures compatible with autophagic vacuolization (Figure 3c and Figure 4d). These findings demonstrate the feasibility of holotomographic live-cell imaging for longitudinal monitoring of OHP-induced morphological axonal injury, while pathway-specific interpretation requires molecular validation. Representative time-lapse holotomographic acquisitions are provided in Appendix A.3 and are presented as dynamic complementary material to the corresponding static figure panels. Appendix A.3 further illustrates the temporal progression of neurite beading, fragmentation, progressive neurite degeneration, cell-body morphological deterioration, and intracellular vacuolar structures compatible with stress-related autophagic vacuolization during the 24–52 h post-treatment imaging interval. Together, these longitudinal recordings and the corresponding figure panels provide complementary static and dynamic evidence supporting RI-based holotomography as a tool to monitor OHP-associated morphological injury in living DRG cultures.

### 3.3. Immunofluorescence and MFI Quantification Show OHP-Associated Changes in NCX2 Signal Across Sensory Neuron Subpopulations

**Figure 5:**
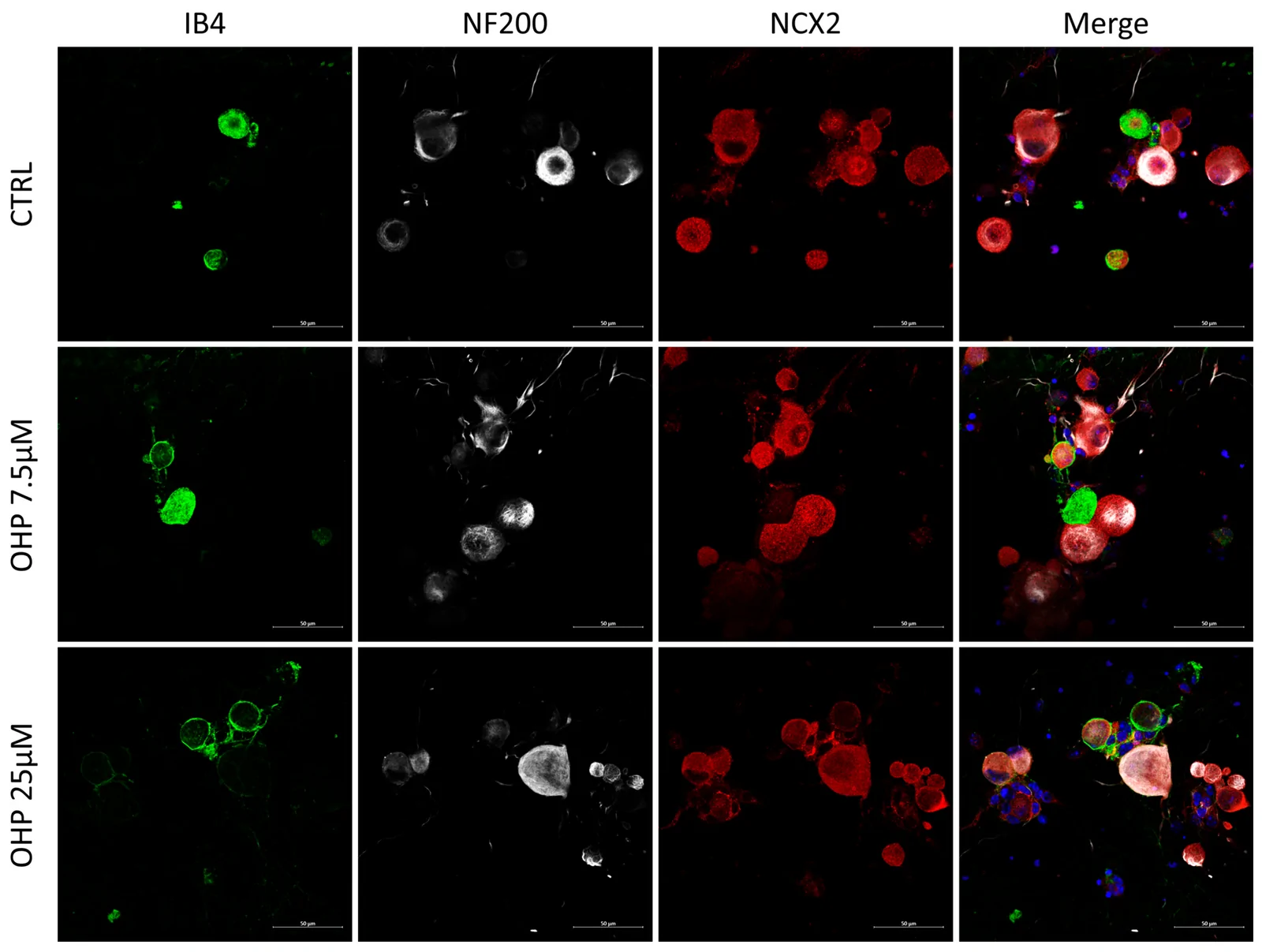
NCX2 expression and fluorescence intensity in IB4-positive and NF200-positive cultured DRG neurons following 24 h of OHP treatment. Representative immunofluorescence images of control cultures and cultures exposed to low-dose (7.5 µM) or high-dose (25 µM) OHP for 24 h, stained for NCX2 (red), IB4 (green), NF200 (white), and DAPI (blue). Mean fluorescence intensity was quantified in IB4-positive and NF200-positive neuronal subpopulations and analyzed using generalized linear mixed-effects modeling. Scale bars: 50 µm.

**Figure 6:**
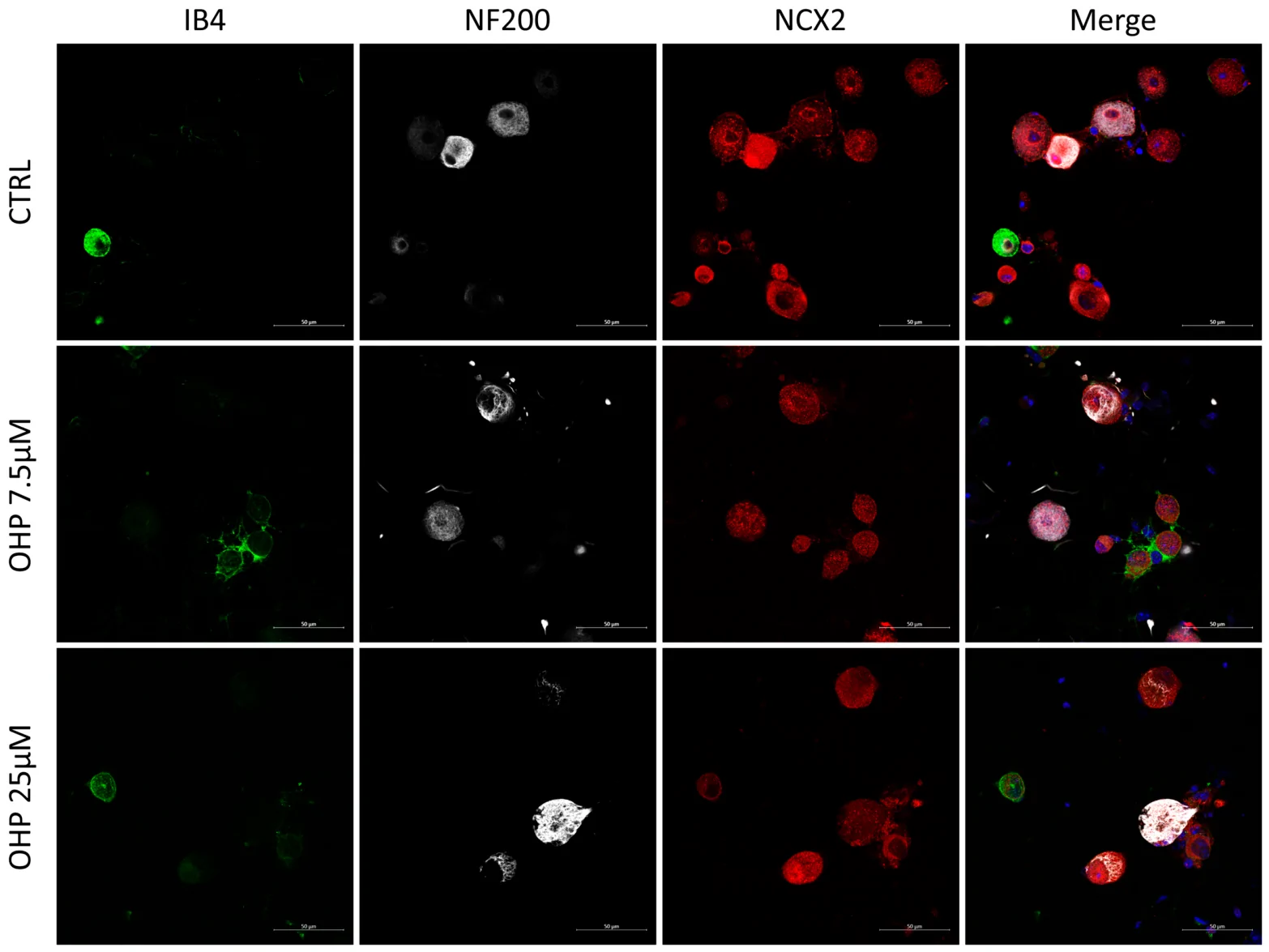
NCX2 expression and fluorescence intensity in IB4-positive and NF200-positive cultured DRG neurons following 48 h of OHP treatment. Representative immunofluorescence images of control cultures and cultures exposed to low-dose (7.5 µM) or high-dose (25 µM) OHP for 48 h, stained for NCX2 (red), IB4 (green), NF200 (white), and DAPI (blue). Mean fluorescence intensity was quantified in IB4-positive and NF200-positive neuronal subpopulations and analyzed using generalized linear mixed-effects modeling. Scale bars: 50 µm.

We quantified mean fluorescence intensity (MFI) in IB4-positive and NF200-positive DRG neuron subpopulations after low- and high-dose OHP exposure at 24 and 48 h. NCX2 was detectable in both neuronal subtypes under control and treated conditions, but quantitative analysis revealed that OHP altered fluorescence intensity in a cell type-, dose-, and time-dependent manner (Figure 5 and Figure 6). In IB4-positive cells, OHP exposure induced a progressive dose-dependent suppression of MFI. At 24 h, low-dose OHP did not significantly alter MFI relative to vehicle control (adjusted *p* = 0.22), whereas high-dose OHP significantly reduced MFI (adjusted *p* = 0.0011). By 48 h, both low- and high-dose OHP produced pronounced decreases in MFI compared with control cultures (adjusted *p* < 0.0001 for both comparisons).

Conversely, NF200-positive cells displayed a dynamic, non-linear MFI pattern after OHP exposure over time (Figure 5 and Figure 6). At 24 h, low-dose OHP significantly decreased MFI (adjusted *p* < 0.0001), whereas high-dose OHP significantly increased MFI (adjusted *p* < 0.0001). By 48 h, NF200-positive neurons showed increased MFI, with both low- and high-dose OHP significantly increasing MFI relative to control cultures (adjusted *p* < 0.0001 for both comparisons). This response was also dose-dependent at 48 h, with high-dose OHP associated with significantly greater MFI accumulation than low-dose OHP (adjusted *p* < 0.0001). Together, these data indicate that OHP was associated with divergent MFI patterns depending on neuronal lineage and exposure time, with IB4-positive cells showing time-dependent MFI suppression and NF200-positive cells showing an initial biphasic shift followed by dose-dependent MFI upregulation.

Although these MFI data provide quantitative support for OHP-induced changes in NCX2-associated fluorescence across IB4-positive and NF200-positive neuronal subpopulations, they do not resolve the subcellular redistribution of NCX2. Specifically, membrane-to-cytoplasmic signal ratios and neurite-specific NCX2 localization were not quantitatively assessed in the present study. Therefore, any interpretation regarding altered NCX2 localization or compartmental redistribution should remain cautious. Future analyses using compartment-based image segmentation, membrane/cytoplasmic intensity ratios, and neurite-specific quantification will be required to determine whether OHP modifies NCX2 distribution within DRG neurons.

### 3.4. SEA0400 Pretreatment Partially Counteracts OHP-Induced Alterations in DRG Neuron Viability and Neurite Elongation

The role of NCX in OIPN was further examined using the same DRG explant model, with neurite elongation and cell viability as readouts of OHP toxicity and SEA0400-mediated neuroprotection.

**Figure 7:**
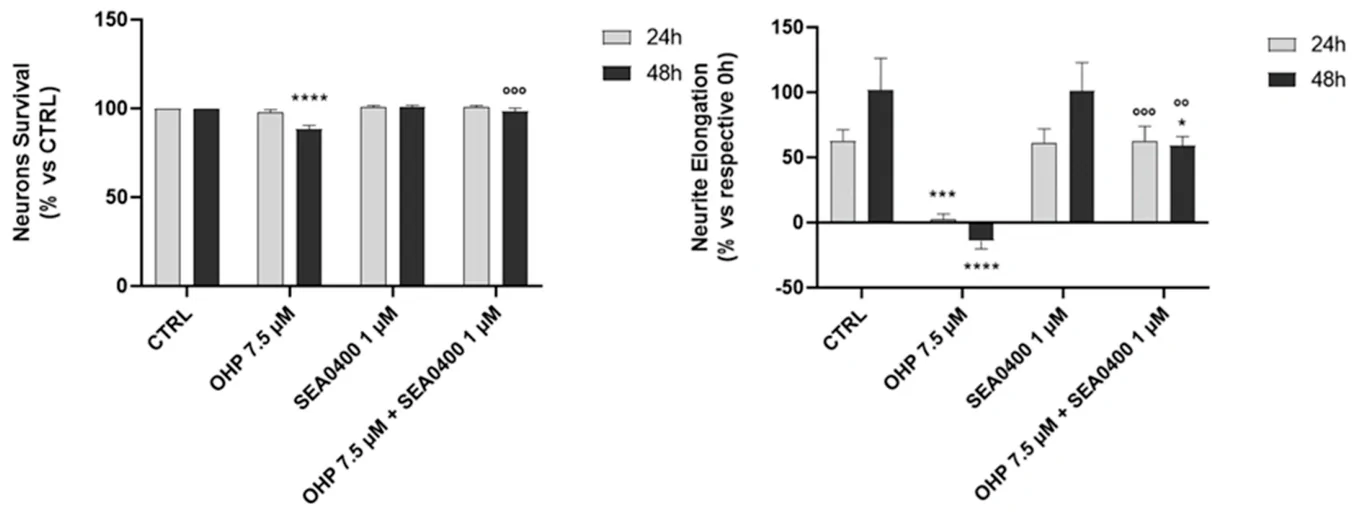
SEA0400 is associated with attenuation of low-dose OHP-induced toxicity in DRG neurons. Histograms show neuronal survival and neurite elongation in control cultures and in cultures treated with 7.5 µM OHP, 1 µM SEA0400, or 7.5 µM OHP plus 1 µM SEA0400 for 24 or 48 h. SEA0400 pretreatment was associated with preserved neuronal viability and partial attenuation of neurite shortening after low-dose OHP exposure. Data are presented as mean ± SEM. Statistical analysis was performed using one-way ANOVA followed by Tukey’s post hoc test; significance values indicate comparisons with untreated control cultures, not comparisons between 24 h and 48 h: * *p* < 0.05, *** *p* < 0.001, and **** *p* < 0.0001 vs. CTRL; °° *p* < 0.01 and °°° *p* < 0.001 vs. OHP 7.5 µM.

As shown in Figure 7, exposure to 7.5 µM OHP caused neurite shortening in DRG neurons after 48 h, while no reduction was observed at 24 h. In contrast, SEA0400 pretreatment (1 µM, 3 h) was associated with preserved neurite elongation at 24 h and partial attenuation of neurite shortening at 48 h. Regarding viability, exposure to 7.5 µM OHP reduced neuronal survival at 48 h, whereas SEA0400 pretreatment was associated with attenuation of this decrease, with viability remaining comparable to control cultures at both 24 and 48 h.

**Figure 8:**
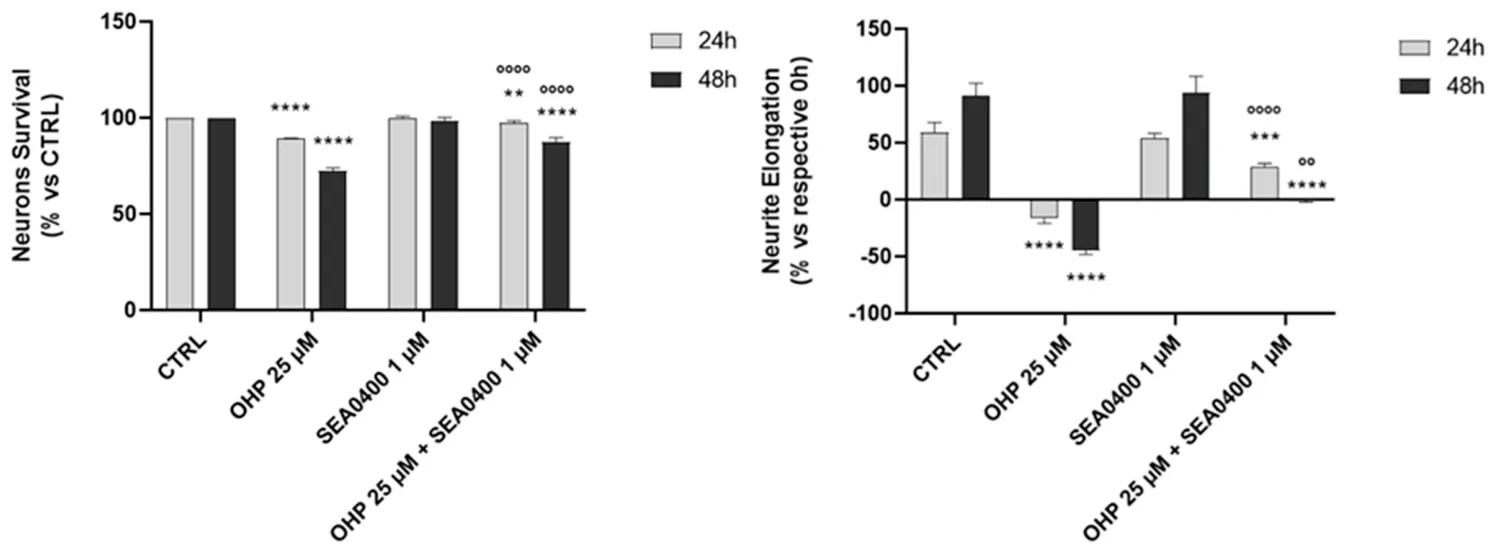
SEA0400 is associated with partial attenuation of high-dose OHP-induced toxicity in DRG neurons. Histograms show neuronal survival and neurite elongation in control cultures and in cultures treated with 25 µM OHP, 1 µM SEA0400, or 25 µM OHP plus 1 µM SEA0400 for 24 or 48 h. SEA0400 pretreatment was associated with partial preservation of neuronal viability and reduced neurite shortening after high-dose OHP exposure. Data are presented as mean ± SEM. Statistical analysis was performed using one-way ANOVA followed by Tukey’s post hoc test; significance values indicate comparisons with untreated control cultures, not comparisons between 24 h and 48 h: ** *p* < 0.01, *** *p* < 0.001, and **** *p* < 0.0001 vs. CTRL; °° *p* < 0.01 and °°°° *p* < 0.0001 vs. OHP 25 µM.

**Figure 9:**
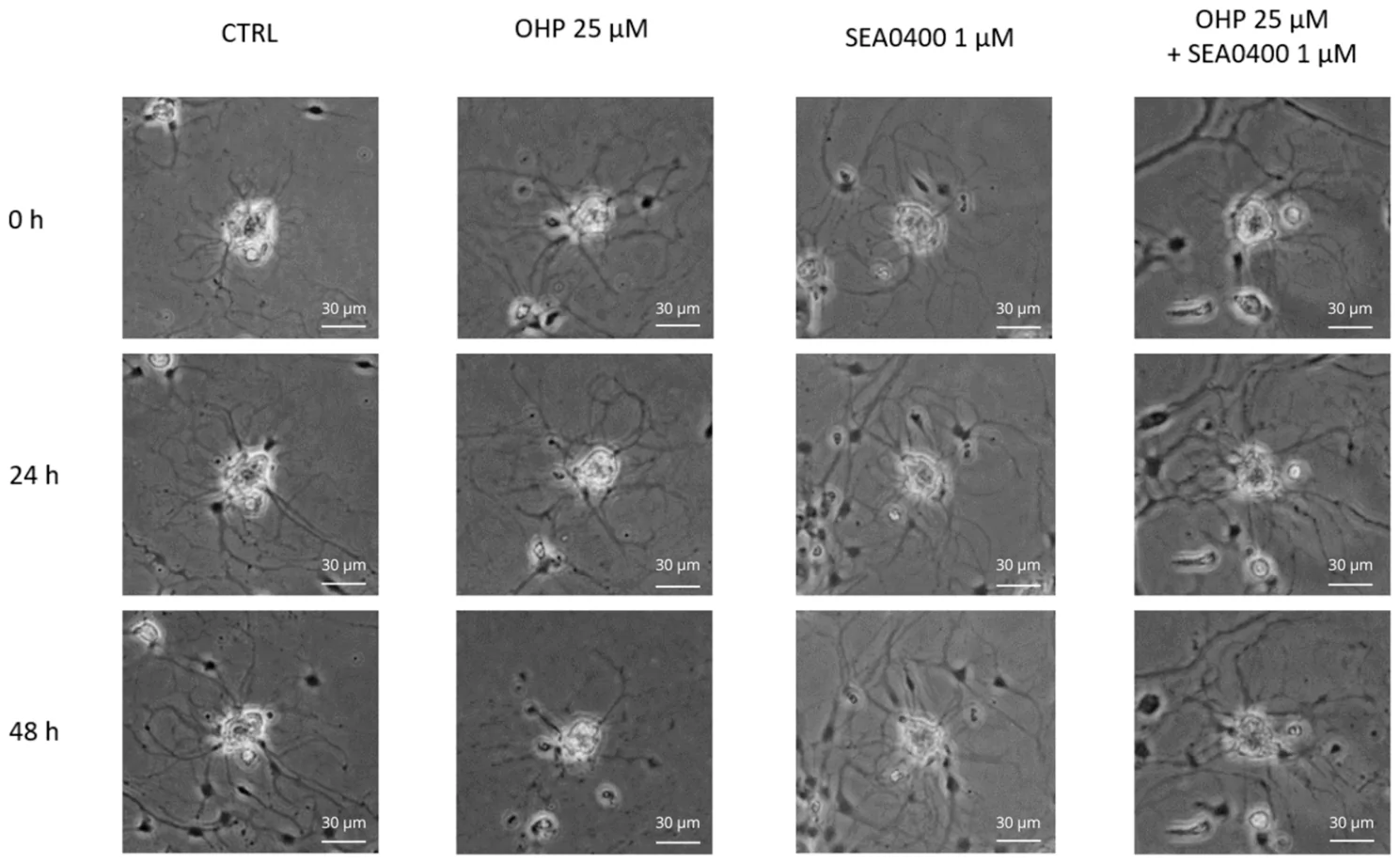
Representative images of DRG neurite elongation after OHP and SEA0400 treatment. Representative images show DRG neurons under control conditions and after treatment with 25 µM OHP, 1 µM SEA0400, or 25 µM OHP plus 1 µM SEA0400 at 24 and 48 h. CTRL, control group; OHP, oxaliplatin-treated cells; SEA0400, SEA0400-treated cells.

As shown in Figure 8, exposure to 25 µM OHP induced marked neurite shortening and reduced neuronal viability at both 24 and 48 h. SEA0400 pretreatment (1 µM, 3 h) was associated with partial attenuation of these effects, with improved neurite elongation and cell survival compared with OHP alone. Representative images illustrating neurite morphology under control, OHP, SEA0400, and OHP + SEA0400 conditions are shown in Figure 9.

### 3.5. 3D Holotomographic Imaging Shows SEA0400-Associated Changes in Lipid Droplet Accumulation After OHP Exposure

**Figure 10:**
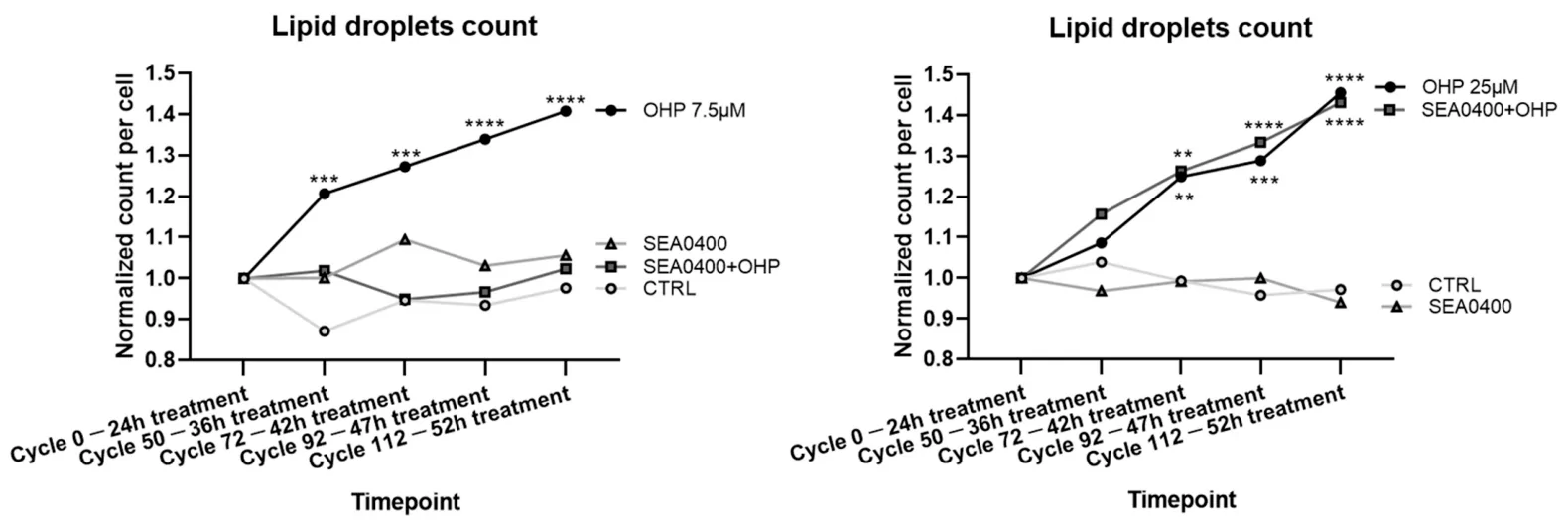
SEA0400 is associated with reduced lipid droplet accumulation induced by low-dose, but not high-dose, OHP. Graphs show lipid droplet count per cell in DRG neurons under control conditions and after treatment with OHP, SEA0400, or SEA0400 plus OHP. Treatment conditions are indicated alongside the corresponding curves. Left: Exposure to 7.5 µM OHP increased lipid droplet number after 52 h, whereas pretreatment with 1 µM SEA0400 was associated with normalization of lipid droplet counts. Right: Exposure to 25 µM OHP increased lipid droplet number after 52 h, and SEA0400 pretreatment was not associated with normalization of this increase. Data are presented as mean ± SEM. Statistical analysis was performed by two-way ANOVA; significance values indicate comparisons with untreated control cultures: ** *p* < 0.01, *** *p* < 0.001, and **** *p* < 0.0001 vs. CTRL.

The role of NCX in OIPN was also explored using lipid droplet count per cell as a label-free holotomographic readout of cellular stress, rather than as a specific marker of OHP neurotoxicity. As shown in Figure 10, lipid droplet number progressively increased after exposure to both low- and high-dose OHP (7.5 and 25 µM), reaching approximately 40% above control levels after 52 h (*p* < 0.0001 vs. CTRL). SEA0400 alone did not alter lipid droplet count compared with control cultures. Pretreatment with SEA0400 (1 µM, 3 h) was associated with normalization of lipid droplet counts after low-dose OHP, maintaining values comparable to CTRL. In contrast, under high-dose OHP exposure (25 µM), SEA0400 pretreatment was not associated with normalization of lipid droplet accumulation, which remained increased by approximately 40% (*p* < 0.0001 vs. CTRL).

### 3.6. Proof-of-Concept Experiments Using siRNA-Mediated NCX2 Knockdown

#### 3.6.1. Quantitative PCR Confirms Partial siRNA-Mediated Reduction of NCX2 Expression

**Figure 11:**
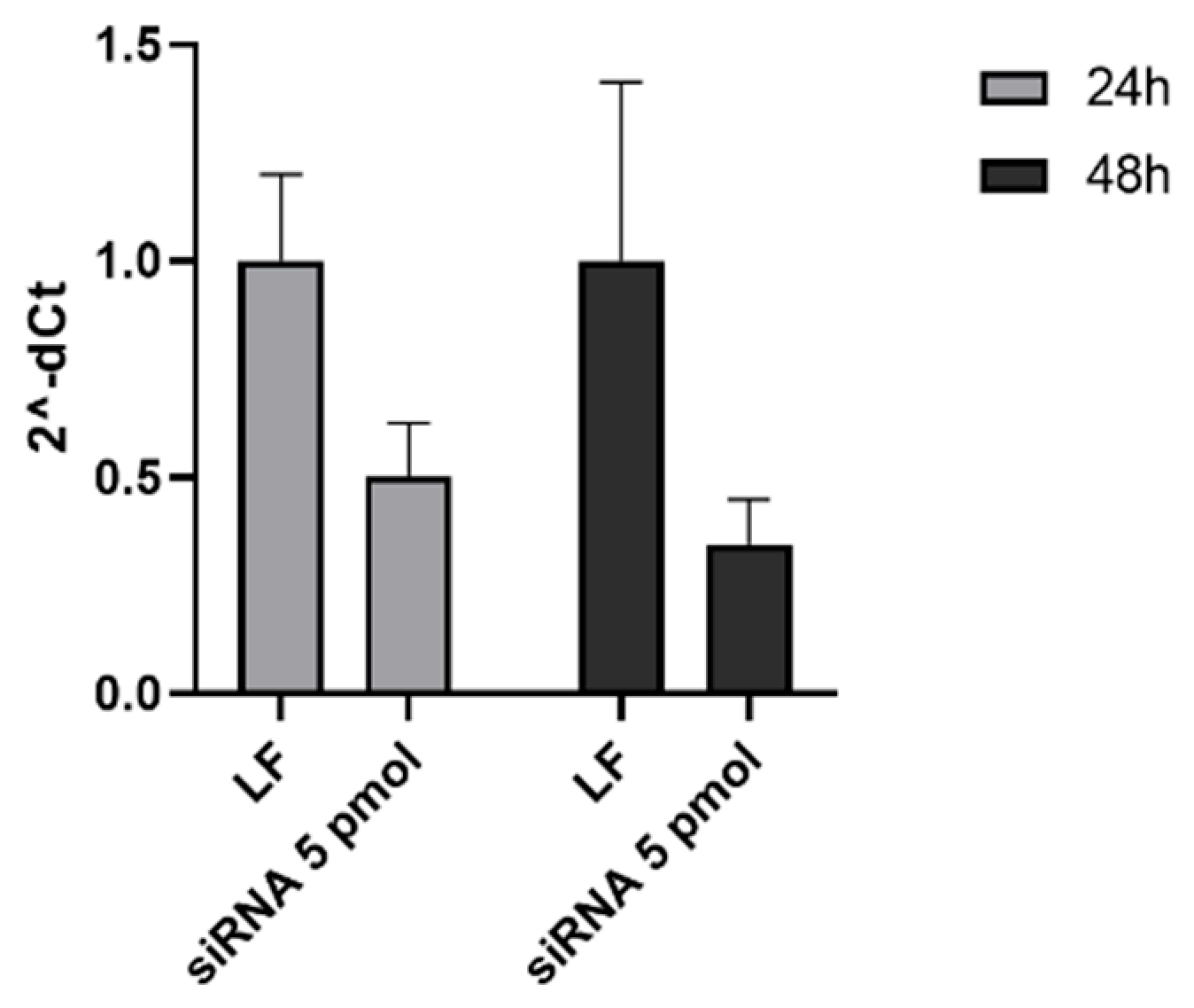
Quantitative PCR validation of siRNA-mediated NCX2 knockdown in DRG cultures. NCX2/Slc8a2 gene expression was measured by quantitative real-time PCR in primary DRG cultures transfected with NCX2-targeting siRNA (5 pmol), selected according to the manufacturer’s datasheet for the commercially validated Ambion Silencer Select siRNA, and compared with Lipofectamine vehicle-treated control cultures. Relative expression levels were normalized to Gapdh and calculated using the 2^−ΔΔCt^ method. siRNA transfection was associated with reduced NCX2 expression relative to vehicle control, consistent with partial target-gene silencing under the experimental conditions used for the neuroprotection assays and with the vendor-reported knockdown efficacy. Data are presented as mean ± SEM; statistical analysis was performed using an unpaired two-tailed *t*-test versus Lipofectamine vehicle control.

To confirm the efficacy of NCX2-targeting siRNA, NCX2 gene expression was evaluated by quantitative PCR in DRG cultures transfected with NCX2 siRNA (5 pmol) and compared with cultures treated with the Lipofectamine vehicle alone. The 5 pmol dose was selected according to the manufacturer’s datasheet for the commercially validated Ambion Silencer Select siRNA and was further supported by our preliminary dose-finding experiments. NCX2 expression was reduced in siRNA-treated cells compared with the vehicle control, indicating partial silencing of the target gene (Figure 11). The magnitude of knockdown observed in our qPCR validation was consistent with the reduction reported by the vendor. This partial reduction is compatible with the neuroprotective phenotype observed while avoiding overt damage to DRG cultures, in agreement with the dose-finding experiments showing no intrinsic toxicity of the selected siRNA condition.

#### 3.6.2. siRNA-Mediated NCX2 Knockdown Partially Counteracts Low-Dose OHP-Induced Alterations in DRG Neuron Viability and Neurite Elongation

**Figure 12:**
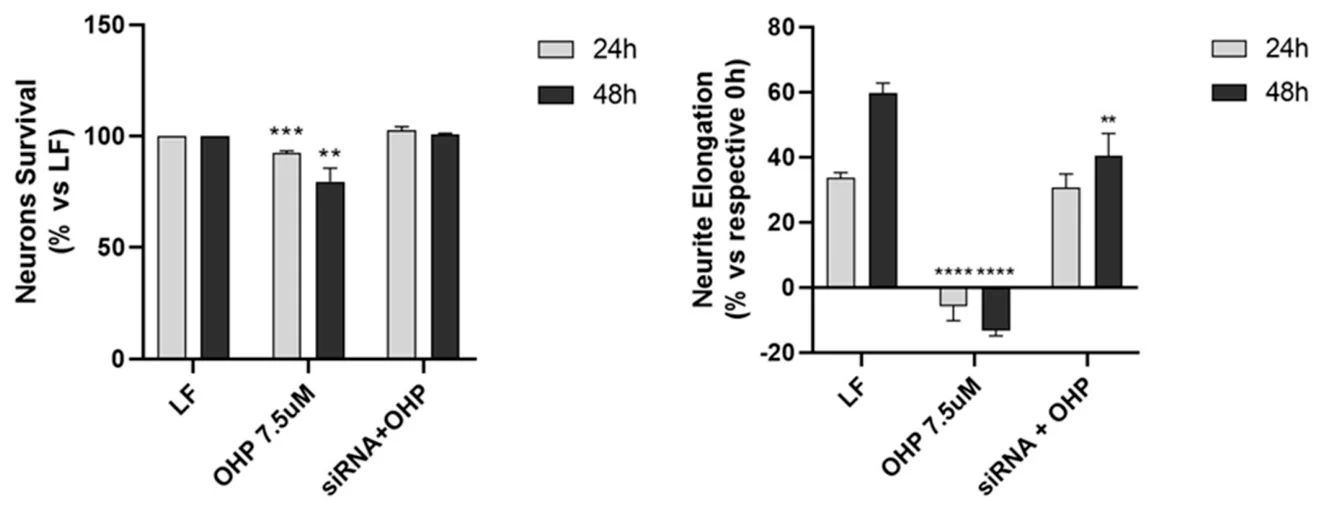
NCX2 knockdown is associated with attenuation of low-dose OHP-induced toxicity in DRG neurons. Histograms show neuronal survival and neurite elongation in vehicle-treated cells, cells exposed to 7.5 µM OHP, cells transfected with NCX2-targeting siRNA, and cells transfected with NCX2-targeting siRNA before OHP exposure. NCX2 knockdown was associated with preserved neuronal survival and attenuation of OHP-induced neurite impairment, particularly at 24 h. Data are presented as mean ± SEM. Statistical analysis was performed using one-way ANOVA followed by Tukey’s post hoc test; significance values indicate comparisons with Lipofectamine vehicle control, not comparisons between 24 h and 48 h: ** *p* < 0.01, *** *p* < 0.001, and **** *p* < 0.0001 vs. Lipofectamine vehicle control.

Low-dose OHP (7.5 µM) induced neurite shortening in DRG neurons compared with vehicle-treated cells, with a mild reduction at 24 h and a more pronounced effect after 48 h of exposure. To evaluate whether NCX2 knockdown was associated with attenuation of OHP-induced neurotoxicity, siRNA transfection was initiated 24 h before OHP treatment. Neurons transfected with NCX2-targeting siRNA (5 pmol) and subsequently exposed to OHP (7.5 µM) showed no significant difference in neurite elongation compared with vehicle-treated cells at 24 h. At 48 h, the toxic effect was only partially attenuated, although neurite growth remained improved compared with OHP treatment alone. Neurons transfected with siRNA in the absence of OHP showed no difference in neurite length compared with vehicle-treated controls. OHP also reduced neuronal viability after both 24 and 48 h of exposure. In contrast, neurons transfected with siRNA before OHP treatment showed no significant reduction in survival compared with vehicle-treated cultures, consistent with a protective effect of NCX2 knockdown against selected OHP-induced neurotoxic endpoints (Figure 12).

#### 3.6.3. 3D Holotomographic Imaging Shows Partial siRNA-Associated Modulation of Lipid Droplet Accumulation After Low-Dose OHP Exposure

**Figure 13:**
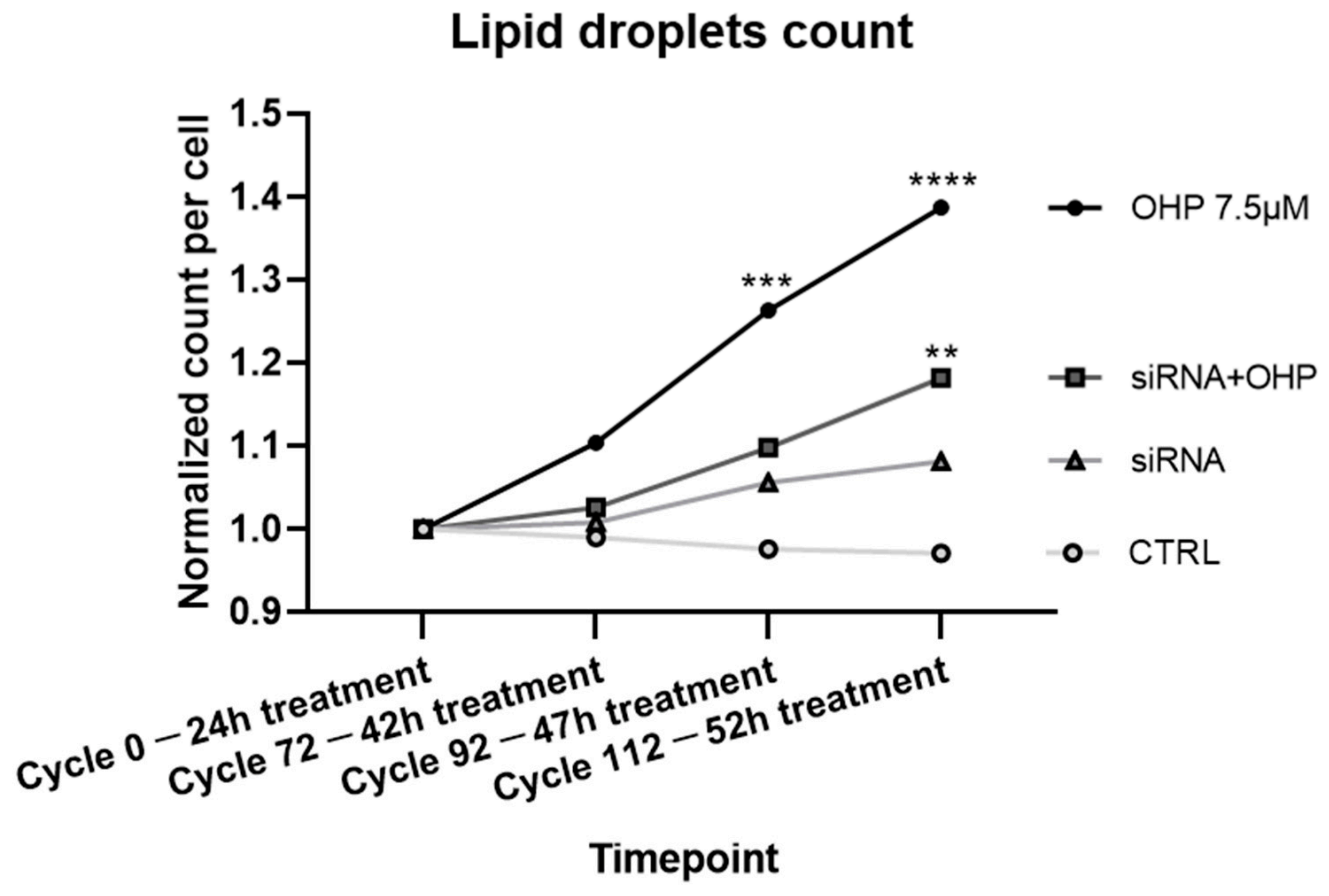
NCX2 knockdown is associated with reduced lipid droplet accumulation induced by low-dose OHP. The graph shows lipid droplet count per cell in DRG neurons under control conditions, after exposure to 7.5 µM OHP, after NCX2-targeting siRNA transfection, and after siRNA transfection followed by OHP exposure. Treatment conditions are indicated alongside the corresponding curves. OHP increased lipid droplet number after 52 h, whereas NCX2 knockdown was associated with a partial reduction in this increase. Data are presented as mean ± SEM. Statistical analysis was performed by two-way ANOVA; significance values indicate comparisons with untreated control cultures: ** *p* < 0.01, *** *p* < 0.001, and **** *p* < 0.0001 vs. CTRL.

The role of NCX2 in OIPN was further explored using lipid droplet count per cell as a label-free holotomographic readout of cellular stress and siRNA-associated modulation, rather than as a specific marker of irreversible neuronal injury. As shown in Figure 13, exposure to low-dose OHP (7.5 µM) progressively increased lipid droplet count per cell, reaching approximately 40% above CTRL levels after 52 h (*p* < 0.0001 vs. CTRL). Pretreatment with NCX2-targeting siRNA (5 pmol, 24 h) did not alter lipid droplet count compared with CTRL cultures. In cells pretreated with siRNA and then exposed to OHP 7.5 µM, lipid droplet accumulation was partially reduced, reaching approximately 20% above CTRL levels (*p* < 0.01 vs. CTRL).

## 4. Discussion

Understanding the cellular and molecular mechanisms that may link acute axonal hyperexcitability to later structural degeneration could support the development of preventive strategies for OIPN [23,36,37]. In this study, our data support the involvement of NCX2 in OHP-induced sensory neuron injury and suggest that NCX2 may represent a candidate target to mitigate OIPN. However, the present findings do not directly demonstrate reverse-mode NCX2 activity or establish a temporal transition from acute hyperexcitability to chronic degeneration. They should therefore be interpreted as functional evidence of NCX2 involvement rather than definitive proof of transporter directionality or disease-stage linkage.

### 4.1. Dose- and Time-Dependent Dynamics of OHP Neurotoxicity

Our in vitro data show that continuous exposure of primary mouse DRG neurons to oxaliplatin induces a progressive dose- and time-dependent impairment of neuronal viability and neurite elongation. At the lowest concentration tested, OHP produced a modest but measurable reduction in neurite length after 48 h, while cell viability remained largely preserved. In contrast, higher concentrations caused marked neurite retraction and significant neuronal loss within the same time frame. This graded response resembles selected aspects of the cumulative and dose-dependent sensory axonal impairment observed clinically, but the present exposure paradigm should be interpreted as a simplified in vitro screening model rather than a direct pharmacokinetic replica of clinical OIPN. These results therefore support the use of primary mouse DRG cultures for investigating neuron-intrinsic mechanisms of OHP toxicity and testing candidate neuroprotective strategies, while requiring validation in clinically aligned in vivo models [38,39].

### 4.2. Differential Subpopulation NCX2 Expression Following OHP Exposure

An important finding of the present study is the differential modulation of NCX2 in distinct sensory neuron subpopulations following OHP exposure. Biologically, IB4 and NF200 distinguish sensory neuron populations that contribute to different components of peripheral sensory function. IB4 labelling identifies predominantly small-diameter, non-peptidergic nociceptors, which are mainly involved in detecting noxious stimuli and transmitting pain-related information, often through unmyelinated or thinly myelinated fibers. In contrast, NF200 labelling identifies large-diameter, neurofilament-rich neurons, which are typically associated with myelinated fibers responsible for proprioceptive, vibration, and mechanosensory signaling [40,41]. Thus, the comparison between IB4-positive and NF200-positive neurons links NCX2-associated fluorescence changes to functionally distinct sensory compartments: a nociceptive compartment mainly related to pain processing and a large-fiber compartment mainly related to proprioception, vibration sense, and sensory coordination. This distinction is relevant for interpreting OHP-induced NCX2 modulation, because changes in these two marker-defined populations may reflect different vulnerabilities or adaptive responses across sensory modalities [42].

Immunofluorescence analysis showed that NCX2 was detectable in both IB4-positive and NF200-positive DRG neurons under control and treated conditions. However, OHP did not affect all neuronal subtypes uniformly. In IB4-positive cells, NCX2-associated MFI showed a progressive dose- and time-dependent decrease, whereas NF200-positive neurons displayed a dynamic pattern, characterized by reduced MFI after low-dose OHP at 24 h, increased MFI after high-dose OHP at 24 h, and significant MFI upregulation after both low- and high-dose OHP at 48 h.

In this context, the reduction of NCX2-associated MFI in IB4-positive nociceptors may reflect a protective downregulation of NCX2 under conditions of sustained OHP-induced depolarization. By limiting NCX2 availability, these neurons could theoretically reduce the likelihood of reverse-mode NCX activity and thereby constrain pathological calcium entry. This interpretation is consistent with the idea that neuronal subpopulations better able to downregulate NCX2 during sustained depolarizing stress may be more resistant to OHP-induced calcium-dependent injury. Conversely, the delayed increase in NCX2-associated MFI observed in NF200-positive neurons may indicate a less effective compensatory response, or a shift from adaptive regulation toward a potentially maladaptive state. However, because the present study did not directly measure membrane NCX2 localization, transporter activity, calcium flux, or subtype-specific neuronal injury, this model should remain hypothesis-generating.

This interpretation is consistent with the known effects of OHP on voltage-gated sodium channels, particularly NaV1.6, which is enriched at nodes of Ranvier and proximal axonal segments of myelinated fibers [23]. OHP-induced sodium channel dysfunction promotes persistent sodium influx and membrane depolarization. In this setting, increased NCX2 availability may initially reflect an attempt to restore ionic homeostasis, while the altered electrochemical gradient could subsequently favor reverse-mode NCX2 activity and calcium overload. The resulting calcium-dependent activation of proteases and organelle stress pathways may then contribute to axonal degeneration in large myelinated fibers. Importantly, the preferential involvement of NF200-positive neurons and large myelinated fibers is consistent with the clinical phenotype of OIPN, in which large-fiber dysfunction and impaired proprioception can manifest as sensory ataxia, with examples including unsteady gait, impaired balance, difficulty walking in the dark or with eyes closed, and reduced manual or gait coordination [8]. By contrast, the reduction of NCX2 signal in IB4-positive nociceptors indicates a distinct response to OHP exposure, but whether this change is compensatory, protective, or secondary to cellular stress remains unresolved and requires further validation.

This subpopulation-specific interpretation remains preliminary because the present MFI analysis quantified fluorescence intensity within neuronal subgroups but did not directly measure NCX2 membrane insertion, transporter activity, or calcium flux. Therefore, the proposed link between increased NCX2 signal in NF200-positive neurons and large-fiber vulnerability should be considered a mechanistic hypothesis requiring confirmation by compartment-specific imaging, functional NCX assays, and electrophysiological or calcium-imaging approaches.

### 4.3. Longitudinal Insight from 3D Holotomographic Live-Cell Imaging

A notable feature of this work is the use of label-free 3D holotomographic live-cell imaging, which enabled continuous and non-invasive monitoring of DRG neurons on the basis of RI signatures [43,44]. By allowing repeated observation of the same cultures over time, this approach reduced the limitations of fixation-, staining-, and endpoint-based analyses and provided a dynamic view of OHP-associated morphological injury. In this setting, holotomography allowed us to follow early and progressive changes in neurite integrity, intracellular organization, and cellular stress-related morphology before overt structural collapse became evident.

Consistent with this longitudinal perspective, OHP exposure was associated with the progressive appearance of neurite fragmentation, vacuolar structures compatible with autophagic vacuolization, morphology-inferred necroptosis-like degeneration, and lipid droplet accumulation [45,46,47]. These changes collectively indicate a pattern of evolving cellular stress rather than an isolated morphological endpoint.

These observations are biologically plausible because OIPN is strongly associated with mitochondrial dysfunction, oxidative stress, and neuronal degeneration. In adult DRG neurons, OHP has been shown to impair mitochondrial respiration and ATP production, while increasing ROS levels and disrupting Ca^2+^ homeostasis [48]. Oxidative stress is also associated with lipid peroxidation, suggesting that altered lipid metabolism may contribute to neuronal damage. In this context, lipid droplets are of particular interest. They are normally poorly detectable in adult DRG neurons, whereas their accumulation can profoundly affect neuronal lipid metabolism and axonal function. For example, Yang and colleagues demonstrated that manipulating triacylglycerol storage and lipid droplet formation in adult mouse DRG neurons alters axonal regeneration [49]. Thus, the increase in lipid droplets observed after OHP treatment may reflect an adaptive response to lipid stress, although excessive or persistent lipid storage could also contribute to neuronal dysfunction. Taken together, the concomitant occurrence of lipid droplet accumulation, neurite degeneration, and neuronal cell death is consistent with a model in which OHP-induced mitochondrial and oxidative stress disrupts lipid homeostasis and contributes to neuronal injury. In addition, OHP can directly impair supportive cell viability and mitochondrial function, potentially further compromising neuronal support [50].

These considerations are important because morphology and refractive-index characteristics alone cannot establish activation of specific cell-death or autophagy pathways. Necroptosis would require molecular validation using markers such as RIPK1, RIPK3, and phosphorylated MLKL, whereas autophagy should be evaluated using LC3-II, p62, and, ideally, autophagic flux experiments performed in the presence and absence of lysosomal inhibitors [45,51,52,53]. Similarly, lipid droplet accumulation should not be considered a specific marker of OHP neurotoxicity or irreversible neuronal injury. Lipid droplets can arise from multiple biological processes, including oxidative stress, mitochondrial dysfunction, altered lipid metabolism, impaired autophagy, and adaptive sequestration of damaged or excess lipids [54,55]. Therefore, in the present study, RI-based imaging is best interpreted as a sensitive label-free approach for monitoring cellular stress-associated morphological changes over time, rather than as definitive molecular identification of necroptosis, autophagy, or neurotoxicity-specific lipid alterations.

### 4.4. Neuroprotection Data Supporting NCX Involvement

The pharmacological experiments indicate that NCX inhibition was associated with attenuation of OHP-induced neurotoxicity in DRG neurons. SEA0400 pretreatment was associated with preserved neuronal viability and partial attenuation of neurite shortening after low-dose OHP exposure. These findings are consistent with our previous observations in embryonic neurons [25]; however, validation in adult DRG neurons was essential to exclude potential bias related to the maturation state of ion channels and transporters [56,57]. SEA0400 also showed measurable attenuation under the more severe 25 µM OHP condition, although the effect was incomplete. This dose-dependent pattern suggests that NCX-sensitive mechanisms may contribute to OHP toxicity, but may be insufficient to prevent injury when the experimental toxic burden is high. Importantly, these findings should not be interpreted as direct pharmacokinetic equivalents of clinical OHP exposure, but rather as evidence that NCX inhibition can modify OHP-induced neuronal stress in this controlled in vitro system.

A limitation of the pharmacological approach is that SEA0400, although widely used as a potent NCX inhibitor, is not selective for NCX2 alone. Therefore, the neuroprotective effects observed after SEA0400 pretreatment cannot be attributed exclusively to NCX2 inhibition. Other NCX isoforms, including NCX1 and NCX3, may also be expressed in DRG neurons and could contribute to the effects observed in this experimental model. In this context, the NCX2-targeting siRNA experiments provide complementary isoform-focused evidence supporting the involvement of NCX2; however, they do not exclude the contribution of other NCX isoforms to the SEA0400-mediated phenotype. We also did not systematically quantify NCX1, NCX2, and NCX3 expression in parallel, nor did we compare pharmacological compounds with different NCX isoform selectivity profiles. Future studies should therefore define the relative expression and localization of all NCX isoforms in DRG neurons and validate the contribution of NCX2 using additional inhibitors and isoform-specific genetic approaches.

The lipid droplet data further refine this interpretation. SEA0400 was associated with normalization of lipid droplet counts after low-dose OHP, whereas it did not normalize the increase observed after high-dose OHP. This divergence argues against a single linear pathway and instead supports the possibility that NCX-dependent mechanisms are one contributor to OHP-induced neuronal stress, especially under less severe toxic conditions. At higher OHP concentrations, additional NCX-independent mechanisms—including mitochondrial dysfunction, oxidative stress, DNA damage, impaired transcription, and programmed axonal degeneration—may become predominant [58]. However, the lower- and higher-dose conditions should be interpreted as experimental stress levels within a simplified in vitro system, not as direct models of single-hit or cumulative clinical OIPN.

The siRNA experiments provide complementary support for the possible involvement of NCX2. NCX2-targeting siRNA was associated with attenuation of low-dose OHP-induced neurite impairment and preservation of neuronal survival, while also partially reducing lipid droplet accumulation. Because these effects were observed using a genetic approach independent of SEA0400, they support the possibility that NCX2 contributes to selected OHP-induced injury endpoints. However, the present data should be interpreted as evidence of NCX2 involvement rather than direct proof of reverse-mode NCX2 activity, since calcium flux and exchanger directionality were not directly measured in this study.

Several limitations of the siRNA approach should also be acknowledged. The qPCR validation confirms a reduction in NCX2/Slc8a2 mRNA expression under the experimental conditions used here, but it does not directly demonstrate reduced NCX2 protein abundance or transporter activity; however, we followed the dosage/protocol as indicated by the vendor datasheet to reach the intended suppression. In addition, although Lipofectamine vehicle control was included and preliminary dose-finding experiments showed no intrinsic effect of the selected siRNA condition on DRG neuron viability or neurite elongation, Lipofectamine alone does not fully exclude non-specific effects related to siRNA transfection. Inclusion of a non-targeting scrambled siRNA control, testing of additional independent NCX2-targeting siRNA sequences, and rescue experiments using siRNA-resistant NCX2 would further strengthen the specificity of the genetic evidence. Therefore, the siRNA results should be interpreted as supportive evidence for NCX2 involvement, while future studies will be required to confirm target specificity at the protein and functional levels.

The present data do not demonstrate a direct molecular interaction between OHP and NCX2. Rather, the proposed relationship is functional and indirect. OHP is known to alter voltage-gated sodium channel kinetics, thereby increasing persistent and resurgent sodium currents, intracellular sodium accumulation, and membrane depolarization [12]. Under these conditions, the electrochemical gradient may favor reverse-mode NCX activity, potentially including NCX2-mediated calcium influx and activation of calcium-dependent degenerative pathways. Therefore, NCX2 should not be interpreted as a direct pharmacological target of OHP, but as a downstream transporter that may participate in OHP-triggered ionic imbalance and axonal degeneration. This interpretation is supported by the effects observed after pharmacological NCX inhibition and siRNA-mediated partial NCX2 knockdown, while also acknowledging that direct measurements of sodium/calcium fluxes and NCX2 transport directionality remain necessary to confirm the proposed mechanism.

Taken together, these findings are consistent with the broader hypothesis that OHP-induced sodium channel dysfunction and sustained depolarization may promote pathological calcium entry through NCX-dependent mechanisms, potentially including reverse-mode NCX2 activity [14,25]. Nevertheless, this mechanism remains inferential in the present study. We did not directly measure intracellular Na^+^ or Ca^2+^ dynamics, membrane potential, NCX-mediated currents, or the direction of NCX transport. Therefore, the protective effects of SEA0400 and NCX2-targeting siRNA indicate that NCX inhibition and partial NCX2 knockdown attenuate OHP-induced neuronal injury, but they do not prove that this protection is specifically mediated by the inhibition of reverse-mode NCX2 activity. Future studies combining live Na^+^/Ca^2+^ imaging, electrophysiological assessment of NCX currents, and manipulation of extracellular Na^+^ and Ca^2+^ gradients will be required to confirm this mechanistic model.

Another limitation is that the present study does not directly assess the acute electrophysiological phase of OIPN. Although OHP-induced axonal hyperexcitability, sodium channel dysfunction, altered action potential firing, and cold-evoked responses are well described in previous studies [10,11,12,13], these endpoints were not measured here. Our experiments focused on later cellular outcomes, including neuronal survival, neurite elongation, lipid droplet accumulation, and morphological degeneration over 24–52 h. Therefore, the current data do not directly demonstrate a temporal or mechanistic transition from acute hyperexcitability to chronic structural degeneration. Instead, they support the involvement of NCX/NCX2 in OHP-induced neuronal injury within this in vitro model. Future studies combining early electrophysiological recordings with longitudinal structural imaging will be required to determine whether NCX2-dependent mechanisms functionally connect acute OHP-induced hyperexcitability to later axonal degeneration.

SEA0400 and NCX2-targeting siRNA were administered before OHP exposure; therefore, the present experiments assessed preventive neuroprotection rather than reversal of established neuronal damage. This distinction is particularly relevant for chemotherapy-induced peripheral neurotoxicity, because, unlike many other neuropathies, the neurotoxic insult is predictable: the timing of chemotherapy initiation is known in advance, and neuroprotective strategies can therefore be started before or at the time of OHP administration. Accordingly, the present findings support NCX/NCX2 modulation as a candidate preventive strategy aimed at limiting the development of OHP-induced neuronal injury, rather than as evidence for nerve regeneration or reversal of established axonal degeneration. Nevertheless, because not all patients develop clinically significant OIPN and because some patients may present only after symptoms have emerged, future studies using post-exposure treatment paradigms will also be valuable to determine whether NCX modulation can attenuate established injury or support regenerative recovery.

### 4.5. Clinical and Translational Perspectives

From a translational standpoint, these findings support further investigation of NCX2 as a potential target for neuroprotective therapies in patients receiving OHP-based chemotherapy, while emphasizing that this proposal remains mechanistic and preclinical. Current clinical interventions for chemotherapy-induced peripheral neurotoxicity are largely symptomatic and offer limited disease-modifying benefits [59]. Consequently, targeting early ionic imbalance may represent a promising preventive strategy; however, because reverse-mode NCX2 activity was not directly measured here, its contribution should be considered a working hypothesis rather than an established mechanism. Future studies will need to determine whether NCX2 modulation can reduce OIPN-relevant injury under clinically representative exposure paradigms and without compromising OHP antitumor efficacy.

A further important limitation concerns the pharmacokinetic relevance of the OHP exposure paradigm used in vitro. In clinical practice, OHP is typically administered as an intravenous infusion over approximately 2 h, after which the concentration of unbound drug declines because of distribution, protein binding, metabolism, and clearance [60]. By contrast, our DRG cultures were exposed continuously to OHP for 48–52 h at nominal concentrations ranging from 7.5 to 50 µM. These conditions do not reproduce the temporal pharmacokinetic profile of clinical OHP administration and should therefore be interpreted as simplified experimental neurotoxic stress conditions rather than direct clinical exposure equivalents. In particular, continuous exposure to higher concentrations, especially 25–50 µM, may induce severe cellular stress and non-specific cytotoxicity and should not be overinterpreted as a faithful model of cumulative clinical OIPN. The clinical relevance of the present findings therefore lies not in the absolute OHP concentrations used, but in identifying NCX2-associated mechanisms that may contribute to sensory axonal injury.

A further limitation is that the present study was conducted in primary DRG neuronal cultures and should therefore be interpreted as a proof-of-concept investigation of direct neuronal responses to OHP. Although this approach is useful for isolating neuron-intrinsic mechanisms of toxicity and neuroprotection, OIPN is a complex disorder involving not only sensory neurons but also Schwann cells, satellite glial cells, macrophages, peripheral nerve terminals, the vascular compartment, the blood–nerve barrier, and central sensory pathways. Moreover, this in vitro model cannot reproduce systemic drug pharmacokinetics, neuroinflammatory responses, sensory behavior, nerve conduction abnormalities, tissue-level platinum accumulation, or the multicellular organization of peripheral nerves and DRG. Accordingly, the present findings require validation in repeated-dose in vivo OIPN models, incorporating behavioral assessments, sensory nerve conduction studies, intraepidermal nerve fiber density, and histological examination of DRG and peripheral nerves [14].

Future in vivo studies should determine whether systemic NCX2 modulation can preserve sensory function in tumor-bearing models without compromising the anticancer efficacy of OHP [38]. Available evidence suggests that NCX2 modulation may be compatible with cancer therapy, although this possibility requires direct experimental validation. Given that oxaliplatin is primarily used in colorectal cancer, it is particularly relevant that reduced NCX expression has been associated with a more favorable prognosis in patients with stage II–IV colon cancer [61]. Moreover, the NCX family has been implicated in tumor progression through its bidirectional control of intracellular Ca^2+^ homeostasis. Under physiological conditions, NCX generally operates in forward mode to extrude excess Ca^2+^; however, features of the tumor microenvironment, including hypoxia, extracellular acidosis, and altered membrane potential, may favor reverse-mode activity, promoting Ca^2+^ influx and activating pro-survival, migratory, and metabolic signaling pathways in cancer cells [62]. Thus, NCX2-targeted neuroprotection may represent a mechanistically grounded strategy to mitigate OHP-induced sensory neurotoxicity while potentially avoiding interference with oxaliplatin antitumor activity [63].

## 5. Conclusions

In conclusion, this proof-of-concept study shows that pharmacological NCX inhibition with SEA0400 and partial NCX2 knockdown by siRNA can partially mitigate OHP-induced injury in primary mouse dorsal root ganglion neurons, as indicated by improved neuronal viability and neurite elongation. Label-free 3D holotomographic imaging provided additional descriptive information on OHP-associated morphological injury, lipid droplet accumulation, morphology-inferred necroptosis-like degeneration, and other cellular stress-related changes. However, the study does not directly demonstrate reverse-mode NCX2 activity, NCX2-dependent calcium influx, a temporal transition from acute hyperexcitability to chronic degeneration, or pathway-specific mechanisms of necroptosis or autophagy.

Therefore, the main conclusion of the present study is limited to the observation that NCX inhibition and NCX2-targeting siRNA attenuate selected OHP-induced neurotoxic endpoints in cultured sensory neurons. Further studies incorporating electrophysiology, Na^+^/Ca^2+^ imaging, molecular pathway validation, isoform-specific approaches, and clinically aligned in vivo OIPN models will be required to define the mechanistic and translational relevance of NCX/NCX2 modulation.

## Data Availability

Data can be found in the Bicocca Open Archive Research Data (BOARD): https://board.unimib.it/datasets/c9z6v6z86b/1 (Alberti, Paola (2 September 2026)), “Holotomographic microscopy—Halting the Reverse Mode: NCX Inhibition as a Neuroprotective Strategy against Oxaliplatin-Induced Peripheral Neurotoxicity”, Bicocca Open Archive Research Data, V2, https://doi.org/10.17632/c9z6v6z86b.2.

## Abbreviations

The following abbreviations are used in this manuscript:

ANOVA	Analysis of variance
AxD	Axonal damage
BSA	Bovine serum albumin
CTRL	Control
DAPI	4′,6-diamidino-2-phenylindole
DMEM	Dulbecco’s modified Eagle medium
DMSO	Dimethyl sulfoxide
DRG	Dorsal root ganglion
FOLFOX	Folinic acid, fluorouracil, and oxaliplatin
IB4	Isolectin B4
Na_V_	Voltage-gated sodium channel
NCX	Sodium–calcium exchanger
NCX2	Sodium–calcium exchanger isoform 2
NDS	Normal donkey serum
NF200	Neurofilament 200
OHP	Oxaliplatin
OIPN	Oxaliplatin-induced peripheral neurotoxicity
PBS	Phosphate-buffered saline
RI	Refractive index
SEM	Standard error of the mean
siRNA	Small interfering RNA
SLC8A2	Solute carrier family 8 member A2
XELOX	Capecitabine and oxaliplatin

## Appendix A

### Appendix A.1. Low-Dose SEA0400 Treatment Does Not Affect DRG Neuron Viability or Neurite Elongation

The potential intrinsic neurotoxicity of SEA0400 was evaluated in vitro using the established DRG explant model from C57BL/6J male mice, with neurite elongation and neuronal viability as primary readouts.

DRG cultures were exposed to SEA0400, a potent NCX inhibitor, at 0.5, 1, or 3 µM for 24 or 48 h. A DMSO control was included at the concentration corresponding to that used for the 3 µM SEA0400 condition. SEA0400 did not induce significant alterations in neuronal viability or neurite elongation at any concentration or time point compared with CTRL cultures, indicating that the doses selected for the neuroprotection experiments were not intrinsically neurotoxic.

### Appendix A.2. siRNA Transfection Does Not Affect Neurite Elongation or Neuronal Survival in Primary DRG Cultures

To exclude potential toxicity associated with the siRNA transfection procedure, primary DRG neuronal cultures were exposed to different doses of NCX2-targeting siRNA (1, 5, or 10 pmol) or to the vehicle alone (Lipofectamine). Neurite elongation and neuronal survival were assessed at baseline and at 24 and 48 h after transfection.

Across all tested siRNA doses, transfected neurons showed no significant alterations in neurite elongation or neuronal survival compared with Lipofectamine-treated controls at either 24 or 48 h (Figure A2). These dose-finding experiments support the use of 5 pmol siRNA for NCX2 knockdown in subsequent neuroprotection experiments.

### Appendix A.3. Holotomographic Video Acquisitions, Nanolive Videos

The videos can be downloaded here: https://board.unimib.it/datasets/c9z6v6z86b/1 (Alberti, Paola (2 September 2026), “Holotomographic microscopy—NCX2 Targeting as a Neuroprotective Strategy against Oxaliplatin-Induced Peripheral Neurotoxicity”, Bicocca Open Archive Research Data, V1, https://doi.org/10.17632/c9z6v6z86b.1).

The video provides a dynamic visualization of prominent OHP-induced morphological changes observed by 3D holotomographic live-cell imaging, including neurite fragmentation, degenerative changes, morphology-inferred necroptosis-like features, and vacuolar structures compatible with autophagic vacuolization.

The video. Live-cell holotomographic imaging of OHP-induced morphological changes in DRG neurons. These videos show primary adult mouse DRG neuronal cultures exposed to OHP at 7.5 or 25 µM. Refractive index-based imaging captures neurite fragmentation and degeneration, morphology-inferred necroptosis-like degeneration, and vacuolar structures compatible with autophagic vacuolization. Images were acquired using a 5 × 5 grid scan, with each grid measuring 90 × 90 µm, every 15 min for 28 h at 7 fps, from 24 to 52 h after treatment initiation.
